# Humoral immune responses mediate the development of a restrictive phenotype of chronic lung allograft dysfunction

**DOI:** 10.1172/jci.insight.136533

**Published:** 2020-12-03

**Authors:** Keizo Misumi, David S. Wheeler, Yoshiro Aoki, Michael P. Combs, Russell R. Braeuer, Ryuji Higashikubo, Wenjun Li, Daniel Kreisel, Ragini Vittal, Jeffrey Myers, Amir Lagstein, Natalie M. Walker, Carol F. Farver, Vibha N. Lama

**Affiliations:** 1 Division of Pulmonary and Critical Care Medicine, Department of Internal Medicine, University of Michigan, Ann Arbor, Michigan, USA.; 2 Department of Surgery, Washington University in St. Louis, St. Louis, Missouri, USA.; 3 Department of Pathology, University of Michigan, Ann Arbor, Michigan, USA.

**Keywords:** Pulmonology, Transplantation, Fibrosis, Mouse models, Organ transplantation

## Abstract

Understanding the distinct pathogenic mechanisms that culminate in allograft fibrosis and chronic graft failure is key in improving outcomes after solid organ transplantation. Here, we describe an F1 → parent orthotopic lung transplant model of restrictive allograft syndrome (RAS), a particularly fulminant form of chronic lung allograft dysfunction (CLAD), and identify a requisite pathogenic role for humoral immune responses in development of RAS. B6D2F1/J (H2-b/d) donor lungs transplanted into the parent C57BL/6J (H2-b) recipients demonstrated a spectrum of histopathologic changes, ranging from lymphocytic infiltration, fibrinous exudates, and endothelialitis to peribronchial and pleuroparenchymal fibrosis, similar to those noted in the human RAS lungs. Gene expression profiling revealed differential humoral immune cell activation as a key feature of the RAS murine model, with significant B cell and plasma cell infiltration noted in the RAS lung allografts. B6D2F1/J lung allografts transplanted into *μMt^–/–^* (mature B cell deficient) or activation-induced cytidine deaminase (*AID*)/secretory μ-chain (*μs*) double-KO (*AID^−/−^μs^−/−^*) C57BL/6J mice demonstrated significantly decreased allograft fibrosis, indicating a key role for antibody secretion by B cells in mediating RAS pathology. Our study suggests that skewing of immune responses determines the diverse allograft remodeling patterns and highlights the need to develop targeted therapies for specific CLAD phenotypes.

## Abstract 2

Humoral immune response activation skews chronic rejection of transplanted lung towards the distinctive phenotype of restrictive allograft syndrome in a murine model of orthotopic lung transplantation.

## Graphical abstract

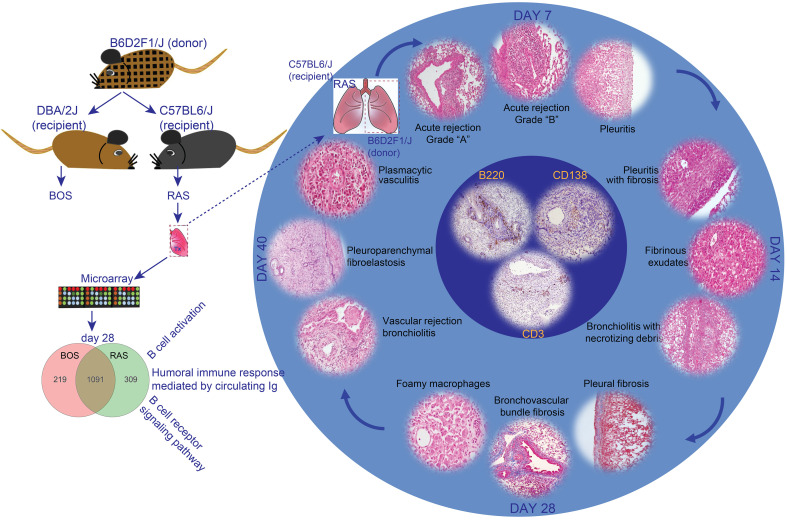

## Introduction

Lung transplantation remains the only viable option for patients with chronic respiratory failure from end-stage lung diseases like cystic fibrosis, idiopathic pulmonary fibrosis, and emphysema. However, long-term survival after lung transplantation continues to be the worst among all solid organ transplants, with a 10-year survival of only 20% (1). The predominant cause of these poor outcomes is the high incidence of chronic graft failure arising from immunologically mediated graft injury and progressive fibrosis termed chronic lung allograft dysfunction (CLAD) (2). Among patients with CLAD, a particularly poor prognosis is associated with a recently characterized subtype designated as restrictive allograft syndrome (RAS) (3). RAS develops in approximately 30% of the patients with CLAD, and it is characterized by a restrictive pattern of decline in lung function and a fulminant course, which leads to respiratory failure and death (3–5).

The histopathological presentations of RAS are more complex and varied than those of bronchiolitis obliterans syndrome (BOS), the other common presentation of CLAD. While bronchiolitis obliterans (BO) or fibrotic remodeling limited to the small airways is a predominant feature of BOS, a spectrum of histologic features have been described in RAS lungs (3, 6, 7). These include more acute presentations of diffuse alveolar damage (DAD) and intraalveolar fibrinous exudates, as well as chronic end-stage fibrosis and pleuroparenchymal fibroelastosis (PPFE) (5, 8–10). Pleural fibrosis extending into the lungs along the interlobular septa, as well as fibrosis emanating from the bronchovascular bundles, is seen pointing to a more fulminant fibroproliferative graft response. Lymphocytic aggregates in the perivascular and peribronchial regions, macrophage accumulation in the airspaces, and presence of B cells have been described (10, 11). This diverse spectrum of pathologies in human RAS specimens, which are obtained at various stages of disease pathogenesis, suggests an evolution from subacute immune-mediated allograft injury and rejection to fibrosis. Patients with persistent donor-specific antibodies (DSA) have been shown to be at a higher risk for RAS (12), and RAS is the dominant form of allograft failure seen in patients with antibody-mediated rejection (AMR) (13, 14). However, investigations of pathogenic mechanisms in this distinctive pleuroparenchymal fibrotic remodeling of allografts have been limited by the lack of a representative animal model (15).

In this study, we describe a murine model of orthotopic single lung transplantation that demonstrates an evolution along the spectrum of histopathological changes that mark RAS in human lung allografts. Investigations of this model highlight immune pathways key to skewing of the remodeling response to RAS and establish an obligatory role for antibody production by B cells in the allograft fibrogenesis in RAS after lung transplantation.

## Results

### Murine orthotopic F1 → parent (B6D2F1/J → C57BL/6J) lung transplants develop allograft fibrosis characteristic of RAS.

**Figure 1:**
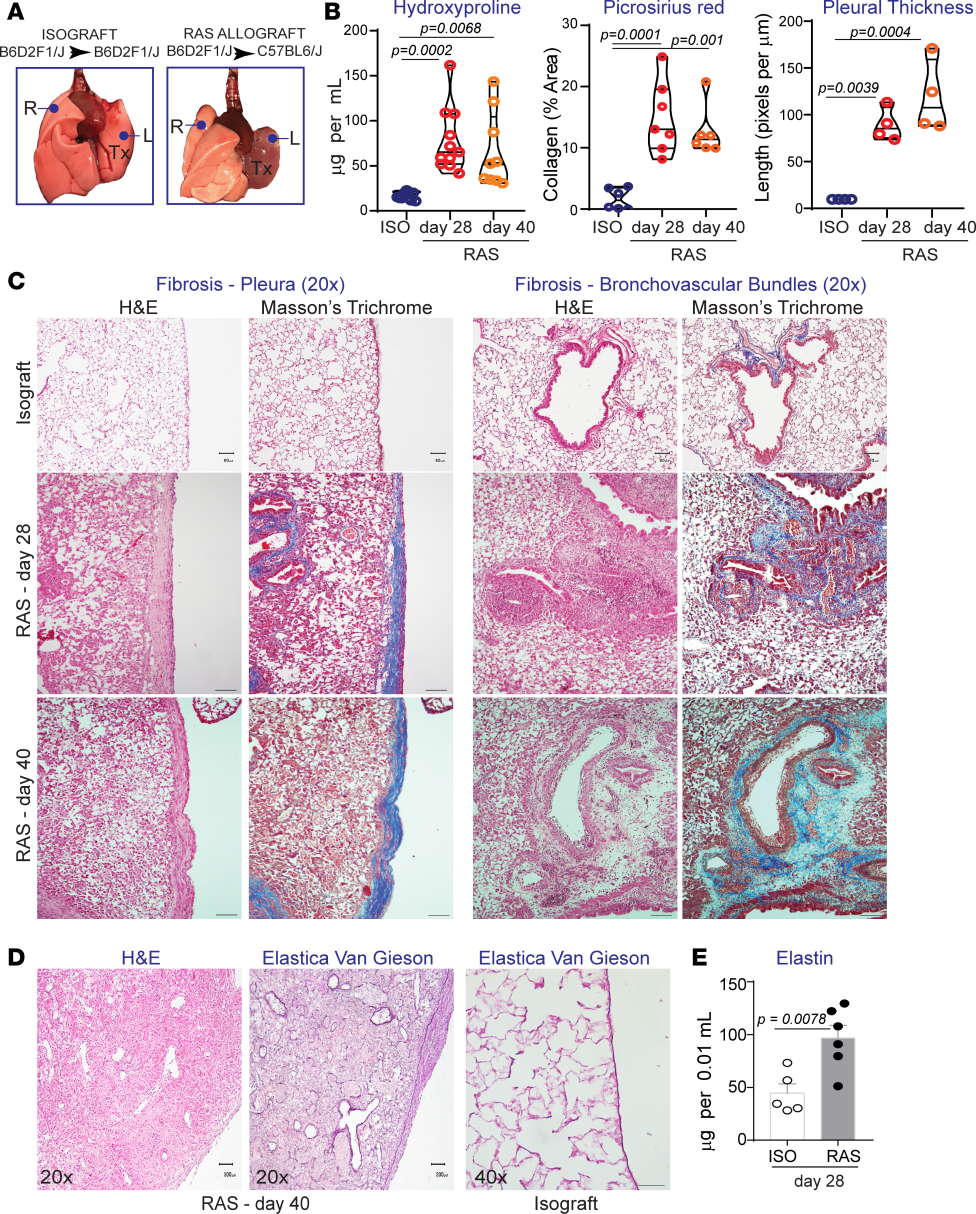
Murine orthotopic lung transplant model of F1 → parent (B6D2F1/J → C57BL/6J) strain combination develop chronic rejection. Single left lung transplants were performed (isografts, B6D2F1/J → B6D2F1/J; RAS allografts, B6D2F1/J → C57BL/6J) and lung explants were either used to obtain lung homogenate (hydroxyproline and elastin assay) or were paraffin embedded for histology (H&E, trichrome and Picrosirius red staining). (**A**) Gross histopathology of control isograft and RAS allograft lungs, showing the transplanted lung on the left (L) and the native recipient lung on the right (R). The isografts were pink and inflated, while the allografts appeared dark and shrunken. (**B**) Quantitative assessment of fibrosis in lung allografts. Hydroxyproline content in graft lung homogenates was measured in triplicates and repeated twice (*n* = 10 isografts, 11 day 28 allografts, 9 day 40 allografts). Collagen staining intensity was measured in tissue sections stained with Picrosirius red using NIH ImageJ. Isografts (day 28), *n* = 6; RAS allografts (day 28), *n* = 7; RAS allografts (day 40), *n* = 6. Pleural collagen was detected in Masson’s trichrome–stained sections, and thickness of this collagen rind was measured using NIH ImageJ. *n* = 4 mice per group. One-way ANOVA with post hoc Dunnett’s. (**C**) Representative sections with H&E and trichrome staining (blue) demonstrating pleural and bronchovascular bundle fibrosis in allografts at day 28 and 40 after transplantation. *n* = 9 transplanted mice were used for histology in each group. Scale bars: 40 μm. (**D**) Elastica Van Gieson staining demonstrating elastin deposition along the pleura and interstitium in a day 40 allograft, compared with the isograft.(*n* = 5 isografts and 6 day 40 allografts). Scale bars: 300 μm. (**E**) Elastin was quantified in the transplant lungs harvested at day 28; *n* = 5 isografts and 6 RAS allografts. Unpaired, 2-tailed *t* test. Data are expressed as mean ± SEM.

Mismatch of immune cells by transfer of T lymphocytes from parent → F1 mice have been used in the fields of graft versus host disease (GVHD), and connective tissue diseases in which different spectra of immune activation and disease severity have been noted depending on the specific parent strain used with the same F1 mouse (16–18). We have previously used this mismatch of F1 and parent mice and have established that transplantation of B6D2F1/J (H2-b/d) F1 lungs into DBA/2J (H2-d) mice leads to the development of pathology characteristic of BO (19). To investigate whether pathology is induced by transplantation of these F1 lungs into the other parent mouse, left lungs from B6D2F1/J (H2-b/d) donor mice were transplanted into C57BL/6J (H2-b) recipients. While isografts (B6D2F1/J → B6D2F1/J) were ventilated and had a normal appearance on gross examination, allogeneic grafts (B6D2F1/J → C57BL/6J) appeared shrunken (Figure 1A). To assess whether there is development of chronic allograft rejection and fibrosis, hydroxyproline assay and morphometric collagen measurements in lung sections stained with Picrosirius red were used (Figure 1B). Significantly higher collagen by hydroxyproline and morphometric analysis was noted in the allografts at both days 28 and 40 after transplant, as compared with the isografts (Figure 1B). Masson’s trichrome collagen staining demonstrated significant pleural thickening and fibrosis, a pathognomonic feature of RAS, in all allografts (Figure 1, B and C). Fibrosis was noted to extend along the subpleural interstitium and the bronchovascular bundles (Figure 1C). Along with pleural and interstitial fibrosis, increased elastin expression and PPFE has been reported in late stages of RAS (8, 10). This led us to evaluate elastin expression in the allografts. We observed increased elastin staining in the pleura and interstitium in approximately one-third of the lung allografts at day 40 after transplantation (Figure 1D). Elastin levels in the homogenized transplanted lungs were also measured by ELISA, with 2-fold higher levels of elastin noted in the allografts compared with the isografts (Figure 1E).

### B6D2F1/J → C57BL/6J allografts demonstrate a spectrum of histopathological characteristics of RAS.

**Figure 2:**
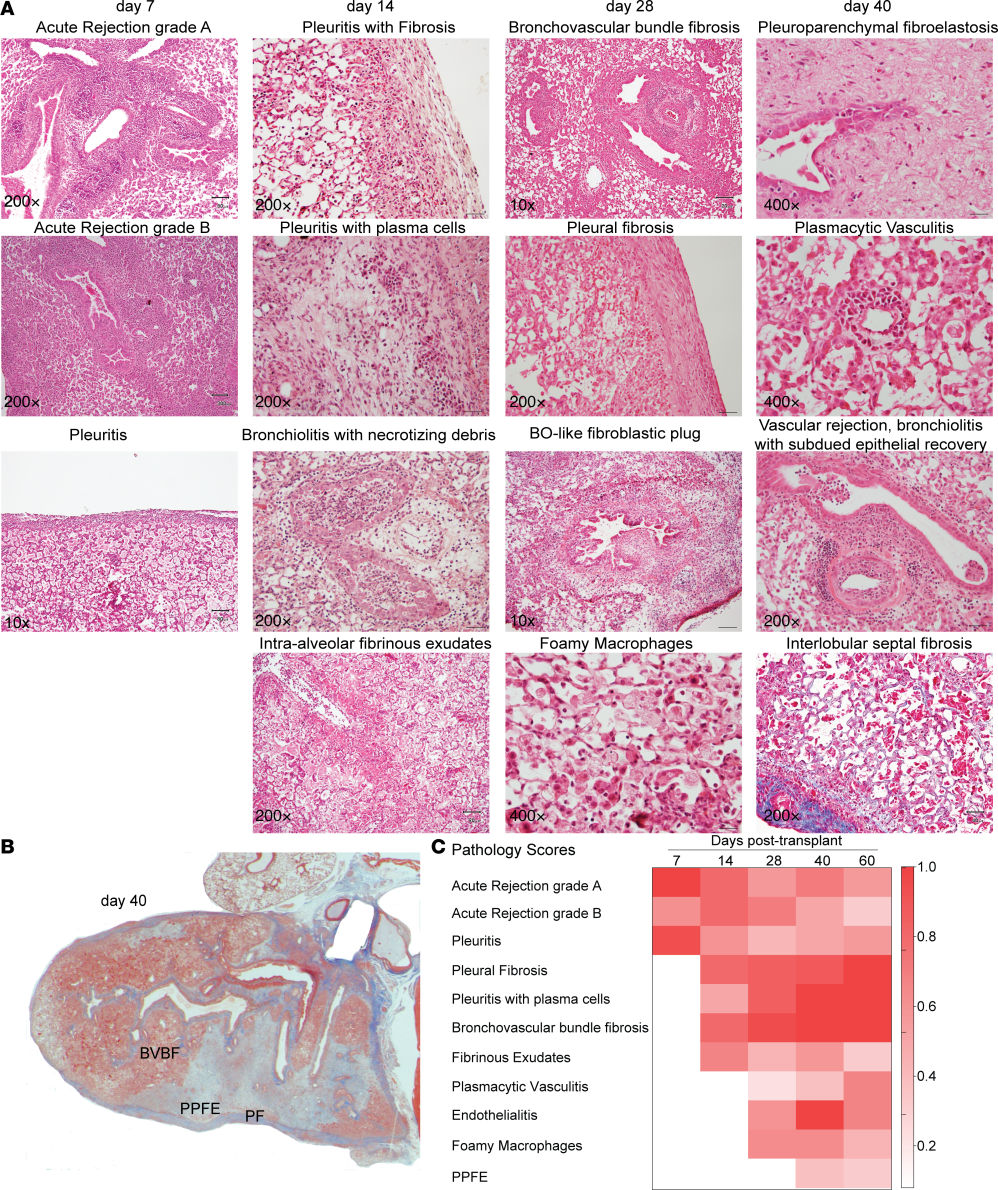
B6D2F1/J → C57BL/6J transplant lungs demonstrate a spectrum of histopathological characteristics of RAS. (**A**) Summary of the histologic characteristics on allograft lungs examined on posttransplant days 7, 14, 28, and 40. Mononuclear cell infiltration of the vessels (acute rejection), airways (lymphocytic bronchiolitis), and pleura (pleuritis) was noted at day 7. Day 14 allografts demonstrated further increase in pleural thickness with plasma cell infiltration and evolving fibrosis. Another prominent feature was development of patches of intraalveolar fibrinous exudates. Fibrosis along the bronchovascular bundles and pleura with occasional fibroblastic plugs in the airway lumen was a key feature at day 28. Alveolar spaces were marked by presence of foamy macrophages. Endothelialitis with evidence of plasma cell infiltration was noted beginning at day 28, and plasmacytic vasculitis marked day 40 allografts. Other findings at this time point included severe pleural fibrosis, along with interlobular septal thickening and fibrosis. Pleuroparenchymal fibroelastosis (PPFE) was noted in some allografts. Bronchovascular bundles demonstrated persistent rejection with epithelial injury. Photomicrographs represent 6–9 mice in each group and were validated by a board-certified pathologist. Lung explants marked for histology at 28 and day 40 overlap with samples shown in Figure 1. New transplants were performed for day 7 and 14 after transplant. Scale bar: 80 μm (original magnification, 200×). (**B**) Photomicrograph of the entire RAS allograft lung with Masson’s trichrome collagen staining (in blue) is shown at day 40. Fibrosis is seen emanating from the pleura and along the bronchovascular bundles. Concomitant histology features included intraalveolar fibrinous exudates and acute rejection. BVBF, bronchovascular bundle fibrosis; PPFE, pleuroparenchymal fibroelastosis; PF, pleural fibrosis. (**C**) Quantitative representation of the histologic characteristics and pathology scores over time after transplant. Average score divided by the highest score at each time point, is presented as a fold-change of 0–1.0 in the heatmap (*n* = 3–9 in each group).

To investigate the temporal evolution of histopathologic changes, lung allografts were studied at various time points after transplantation (days 7, 14, 28, 40, and 60). Histologic patterns were identified and scored by a pulmonary pathologist using a scoring algorithm as described in Methods. Representative images are shown in Figure 2, A and B. Quantitative scoring of the severity of pathologic features is presented as a heatmap in Figure 2C. Percentage incidence based on absence or presence of a specific histologic feature is shown in Supplemental Table 1 (supplemental material available online with this article; https://doi.org/10.1172/jci.insight.136533DS1).

As shown in Figure 2A, the predominant finding at day 7 was the presence of moderate acute rejection with perivascular and peribronchial lymphocytic infiltration. Mild cellular infiltration was also noted in the pleura. By day 14, progression of pleuritis with mesothelial hyperplasia and plasma cell infiltration was evident. Another key histology feature noted at this time point was the presence of patchy fibrinous exudates in the alveoli, characteristic of acute fibrinous pneumonia. Persistent acute cellular rejection with lymphocyte infiltration surrounding blood vessels and airways continued over time, with some lungs also demonstrating distinct clusters of lymphoid cells at day 28. Endothelialitis with infiltration by lymphoplasmacytic infiltrate and endothelial cell damage was noted to be a prominent feature at this time point (Supplemental Figure 1). Another feature of day 28 histology was the appearance of foamy macrophages in the alveoli, concomitant with a decrease in fibrinous exudates. Decreasing cellularity with increasing fibrosis was noted in the pleural space, as evidenced by pale acellular expansion. Fibrotic expansion was also evident along the bronchovascular bundles by day 28. By day 40, all transplanted lungs demonstrated pleural fibrosis, along with peribronchial fibrosis. Along with substantial fibrosis, acute rejection and areas of fibrinous exudates were still noted in the majority of the grafts, demonstrating presence of multiple histologic patterns at a given time point (Figure 2B). Plasma cell infiltration of the pleura and the interstitium persisted at day 60, which was associated with further increase in fibrosis in the pleural space. Thus, B6D2F1/J → C57BL/6J allografts demonstrated the spectrum of characteristic histologic patterns that have been described in human RAS lungs (5, 8–10).

### Gene expression profiling reveals differential humoral immune cell activation as a key feature of the RAS murine model.

**Figure 3:**
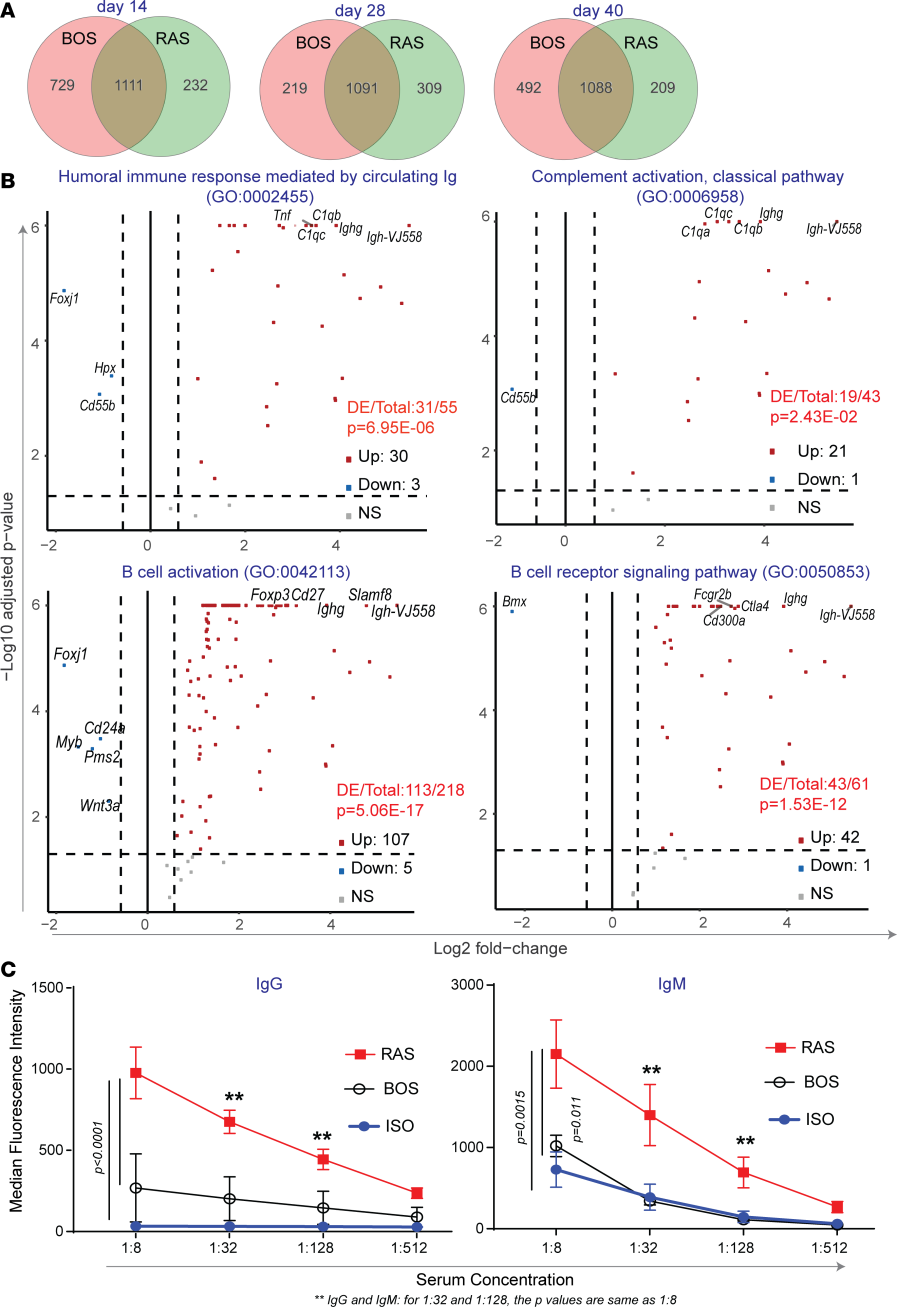
Gene expression signatures reveal differential humoral immune responses unique to RAS allografts. (**A**) Comparisons of significantly enriched GO terms from microarray analyses for RAS and BOS allografts indicated both unique and overlapping affected biological functions. (**B**) Volcano plots showing gene expression impacts for day 28 RAS allografts for the 4 GO terms of interest; dashed lines correspond to absolute fold difference of –1.5 and 1.5, and labels indicate the top 5 of all significantly upregulated genes (red dots) and downregulated genes (blue dots). Total number of differentially expressed (DE) genes and *P* value for each GO term are also reported in Table 1. *n* = 4 per group per time point. (**C**) Serum alloantibody titers of donor-specific IgG and IgM antibodies in day 28 isografts and RAS and BOS allografts. *n* = 4–8 per group. Values are expressed as mean ± SEM. One-way ANOVA with post hoc Bonferroni. **P < 0.01.

The disparate pathology of the B6D2F1/J → C57BL/6J combination, which had histopathologic features of RAS compared with the previously described BO pathology noted in B6D2F1/J → DBA/2J allografts (19, 20), led us to investigate the global gene differences between the 2 models over time by using Affymetrix microarray analyses. Data collected previously from the B6D2F1/J → DBA/2J allografts (BOS, unpublished observations) were normalized and analyzed, along with expression data from B6D2F1/J → C57BL/6J allografts (RAS). We compared biological processes gene ontology (GO) enrichments between the 2 models at days 14, 28, and 40 (Supplemental Table 2). Venn diagrams demonstrating the overlap between significantly enriched GO terms in each of 2 experimental conditions is shown in Figure 3A and Supplemental Table 2. The top GO terms, ranked by significance, which were enriched in the RAS model and not the BOS model for each time point, are presented in Table 1. Humoral immune responses mediated by circulating immunoglobulins was the top GO term at day 14 after transplantation, with B cell signaling and antigen processing related pathways showing preponderance at this stage. Phagocyte recognition, angiogenesis, vascular development, muscle development, and complement activation were among the top GO terms ranked by *P* value in RAS at day 28, consistent with the observed macrophage infiltration and fibrotic remodeling at this time point. Phagocyte recognition continued to be among top enriched GO terms in RAS at day 40. GO terms related to humoral immune responses, B cell activation, B cell receptor signaling, and complement activation demonstrated statistically significant upregulation of gene expression at all 3 time points in the RAS model (Table 2). Volcano plots for differential expression data from day 28 for these key GO terms are shown in Figure 3B. To further investigate humoral responses, we measured serum levels of DSA in isografts (B6D2F1/J → B6D2F1/J) and in BOS (B6D2F1/J → DBA/2J) and RAS (B6D2F1/J → C57BL/6J) lung allografts. High levels of donor-specific serum IgM and IgG were noted in RAS transplants. These levels were significantly higher than those noted in both isograft and BOS serum samples. No significant increase above isografts was noted in BOS samples (Figure 3C).

### Infiltration with B cells and plasma cells characterizes lung allografts in a murine RAS model.

**Figure 4:**
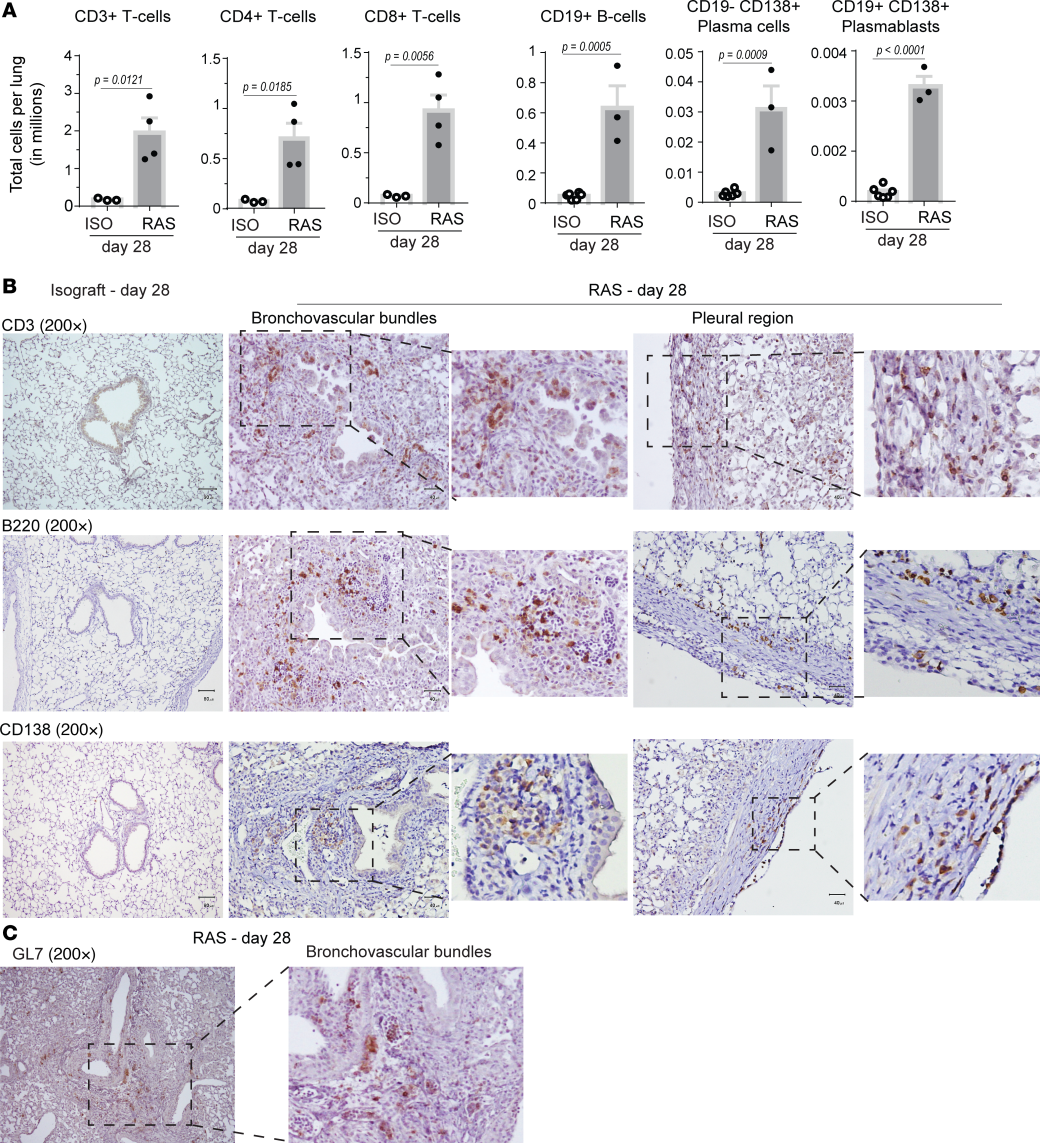
Immunophenotyping and localization of infiltrating cell populations in RAS allografts. (**A**) Single cell suspensions of lung isografts (*n* = 3–6) and allografts (*n* = 3–4) at day 28 after transplant were immunostained and analyzed by flow cytometry to quantitate infiltrating T cells (CD3^+^, CD4^+^, CD8), B cells (CD19^+^), plasma cells (CD19^–^CD138^+^) and plasmablasts (CD19^+^CD138^+^). Data are shown as mean ± SEM, tested using unpaired *t* test. (**B**) Histochemical immunostaining for CD3^+^ (T cells), B220^+^ (B cells), and CD138^+^ (plasma cells) was performed on tissue sections from transplanted lungs marked for histology (*n* = 3 isografts; 5–7 allografts). Representative images demonstrating immune cell infiltration along the bronchovascular bundle and the pleura in day 28 RAS allografts are shown. (**C**) Staining for germinal center marker GL7^+^ cells in a RAS allograft at day 28 is shown. *n* = 5 per group. Scale bars: 40 μm.

Flow cytometry was used to investigate the infiltrating immune cell populations in the RAS allografts (Figure 4A). Increases in both CD4^+^ and CD8^+^ T cells were noted in allografts, as compared with isografts. We also observed significantly higher numbers of CD19^+^ B cells, plasma cells (CD19^–^CD138^+^), and plasmablasts (CD19^+^CD138^+^) in the allografts. Immunostaining with anti-CD3 antibody confirmed T cell infiltration at the bronchovascular bundles (Figure 4B). T lymphocytes were the predominant cell in the lymphoid aggregates, and T cell infiltration of the pleura was also noted. Immunostaining for B220 demonstrated clusters of B220^+^ B cells in the sub-bronchial location in close proximity to the smooth muscle bundles and the mesenchymal cells. B cell infiltration was also noted in the subendothelial and subpleural spaces on day 28 (Figure 4B). CD138^+^ plasma cells were noted predominantly along the bronchovascular bundles and in the pleura (Figure 4B). GL7^+^ expression, a marker characteristic of germinal centers, was also noted in the cellular aggregates localized in the peribronchial region of the RAS day 28 allografts (Figure 4C). To investigate if this evidence for humoral cell activation is unique to RAS, infiltrating B cell population in the allograft were compared between the BOS and RAS models. Significantly lower CD19^+^, CD19^+^CD22^+^, and CD19^+^CD138^+^ B cell populations were noted in the BOS model as compared with RAS allografts by flow cytometry (Supplemental Figure 2A). Immunostaining with CD138 revealed a stark difference between the 2 allografts, with no significant plasma cell infiltration in the BO lungs (Supplemental Figure 2B).

### Requisite role of humoral immune responses in pathogenesis of RAS.

**Figure 5:**
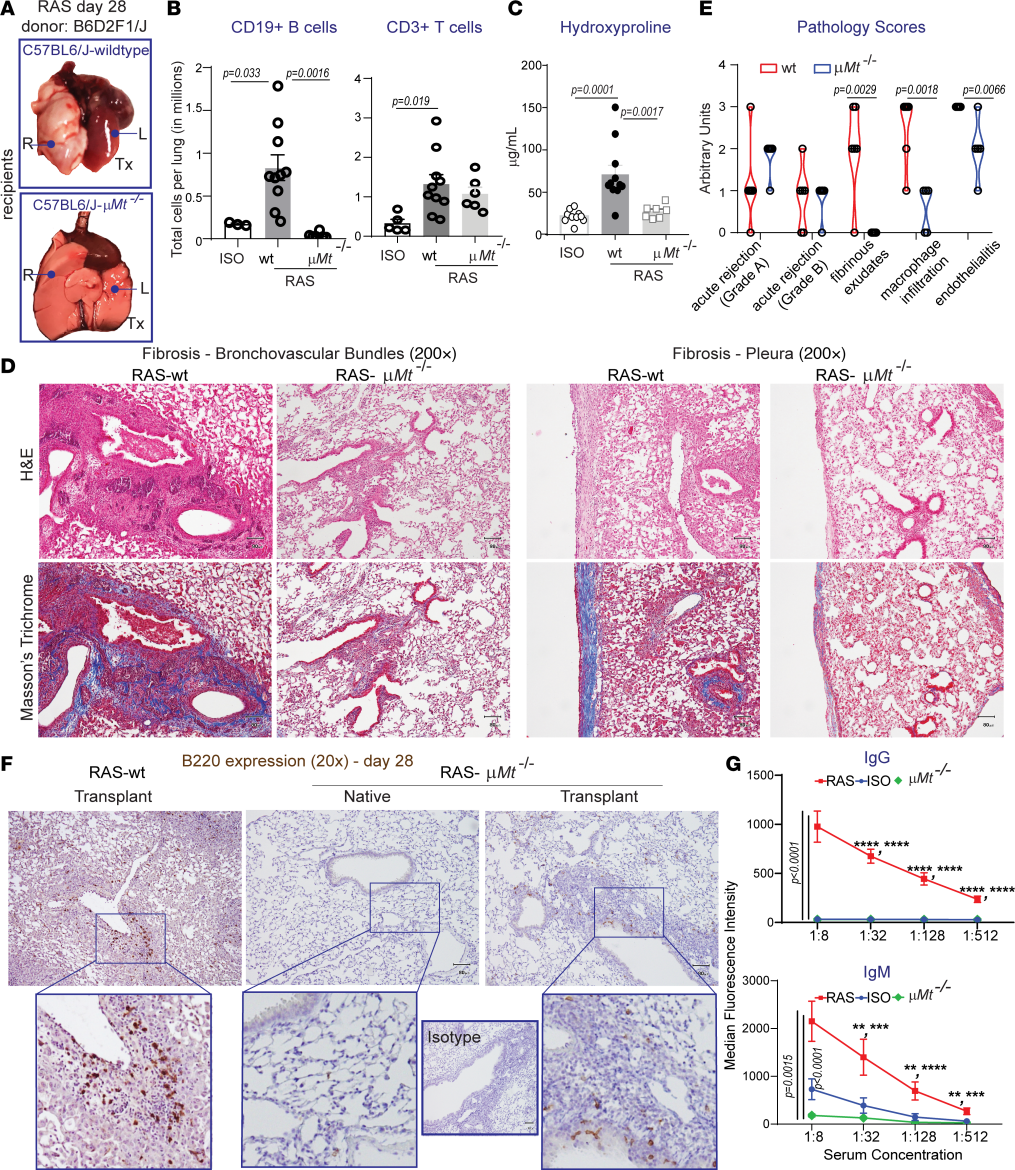
Requisite role for mature B cells in chronic lung allograft rejection leading to RAS. (**A**) Gross histopathological images of *μMt^–/–^* allografts (B6D2F1/J → *μMt^–/–^* C57BL/6J) compared with control allografts (B6D2F1/J → C57BL/6J). Left lung (L), allograft; right lung (R), native lung. (**B**) CD19^+^ B cells and CD3^+^ T cells were analyzed by flow cytometry in lungs of isografts, WT RAS and *μMt^–/–^* RAS allograft recipients. *n* = 3–5 isografts, 10 RAS allografts and 6 RAS allografts with *μMt^–/–^* recipients. Data are shown as mean ± SEM, tested using 1-way ANOVA and Bonferroni. (**C**) Collagen content quantitation by hydroxyproline assay. *n* = 10 isografts, 11 RAS allografts and 7 RAS allografts with *μMt^–/–^* recipients. Data are shown as mean ± SEM, tested using 1-way ANOVA and Bonferroni. (**D**) Compared with RAS lung allografts, histopathological images of *μMt^–/–^* lung allografts show less fibrosis in the bronchovascular bundles and the pleura. Photomicrographs are representative images from 5 mice. Scale bars: 40 μm. (**E**) RAS associated histologic patterns were scored by a blinded pulmonary pathologist in *μMt^–/–^* and WT RAS allografts on a scale of 0–3. Pathology scores are expressed as median along with all data points; *n* = 6 each, and significance was tested using Holm-Šidák method. (**F**) B cells in the allograft of *μMt^–/–^* recipient mice were evaluated by IHC staining with anti-B220 antibody. Staining in RAS allografts as characterized in Figure 3 is shown for comparison. Scale bars: 40 μm. (**G**) Donor-specific IgG and IgM levels measured in the serum derived from isograft, WT RAS allografts, and RAS allograft in *μMt^–/–^* recipients. Serum samples used for analysis of isografts and RAS allografts were also used for analysis displayed in Figure 3C. *n* = 4–8 mice per group. Data are shown as mean ± SEM, tested using 1-way ANOVA and Bonferroni. **P = 0.01, ***P < 0.001, ****P < 0.0001.

To further elucidate the role of B cells in the development of RAS pathology and lung allograft fibrogenesis *μMt^–/–^* (mature B cell deficient) recipient mice were used. B6D2F1/J lung allografts transplanted into *μMt^–/–^* C57BL/6J mice were compared with B6D2F1/J allografts transplanted into WT C57BL/6J mice at day 28 after transplantation. Gross examination demonstrated ventilated allografts in *μMt^–/–^* C57BL/6J mice (Figure 5A). Flow cytometry confirmed decreased B cells in the lung allografts into *μMt^–/–^* C57BL/6J recipient mice, as compared with the RAS allografts, but no significant difference was noted in the number of CD3^+^ T cells between the 2 groups (Figure 5B). Hydroxyproline assay was used to compare total collagen expression in isografts, allografts transplanted into WT recipients, and allografts transplanted into *μMt^–/–^* hosts. Significantly lower levels of collagen were noted in allografts from *μMt^–/–^* recipients as compared with the WT recipient with levels comparable with those in the isografts (Figure 5C). Trichrome staining confirmed attenuated allograft fibrosis in allografts placed into *μMt^–/–^* recipients, with decreased pleural as well as interstitial, peribronchial, and perivascular collagen expression (Figure 5D). Other RAS-associated histologic patterns were scored by a blinded pulmonary pathologist in *μMt^/–^* and WT RAS allografts using a scoring algorithm described in Methods. *μMt^–/–^* C57BL/6J recipients demonstrated significant reduction in fibrinous exudates, macrophage infiltration, and endothelialitis with no significant difference noted in acute rejection scores (Figure 5E). A conspicuous feature was the presence of preserved endothelium in *μMt^–/–^* recipients and the absence of subendothelial plasma cell aggregates despite significant perivascular T cell infiltration (Supplemental Figure 1). Immunostaining for B cells revealed only some scattered B220^+^ cells in allografts that had been transplanted into *μMt^–/–^* mice (Figure 5F). Those B cells in the allografts into *μMt^–/–^* recipients were confirmed to be of donor origin by H2-d staining and flow cytometry (data not shown). Consistent with previous reports from our laboratory (21), we did not observe donor-specific IgM or IgG antibodies in the serum of *μMt^–/–^* allograft recipients (Figure 5G).

**Figure 6:**
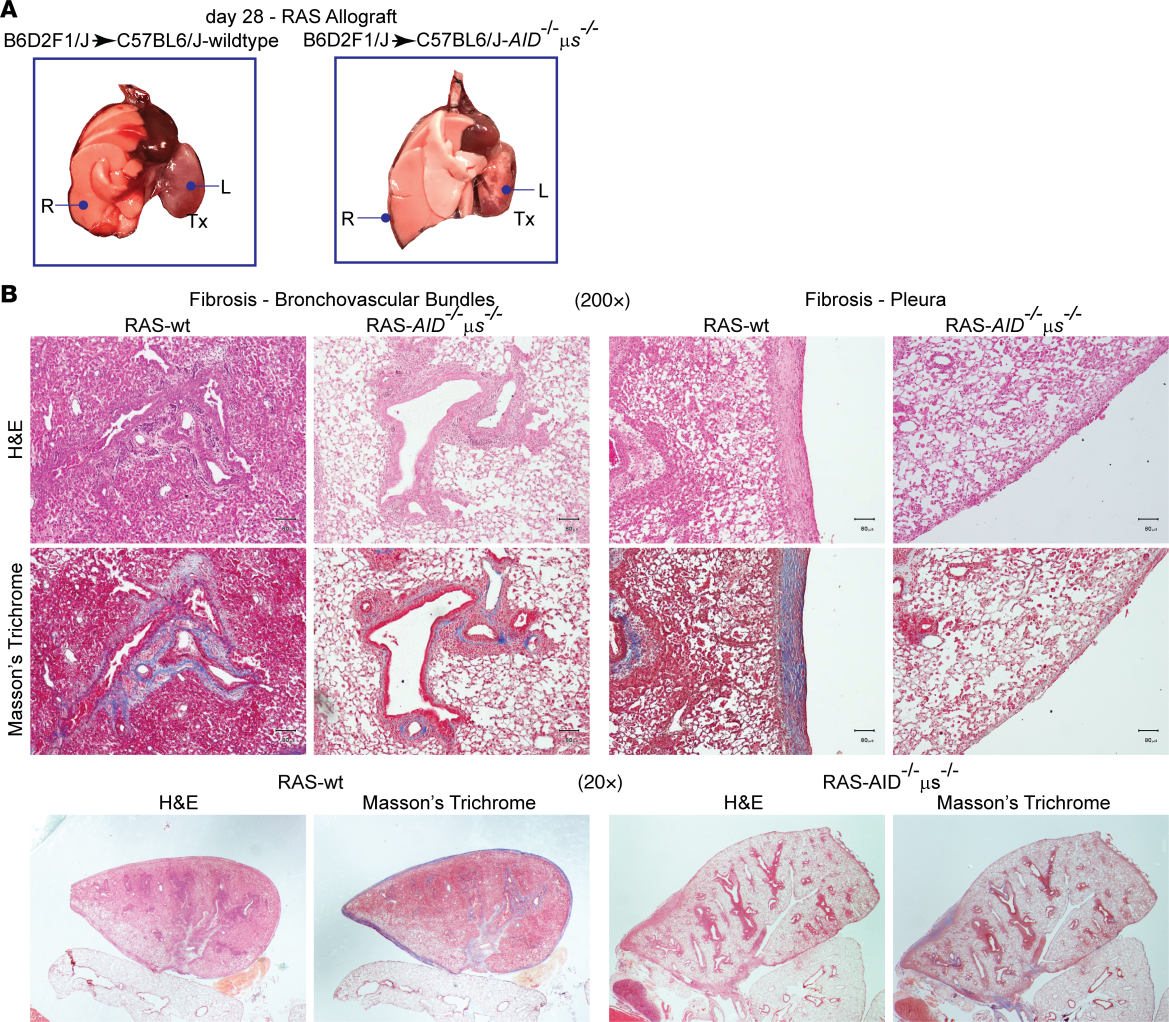
Role of secretory immunoglobulins in the pathogenesis of RAS. (**A**) Gross images of *AID*^–/–^ *μs*^–/–^ allografts (B6D2F1/J → *AID*^–/–^*μs*^–/–^ C57BL/6J) compared with WT allografts (B6D2F1/J to C57BL/6J). Left lung (L), allograft; right lung (R), native lung. (**B**) H&E and trichrome staining of allografts transplanted into WT and *AID*^–/–^ *μs*^–/–^ recipients. Photomicrographs are representative of at least 5 different transplanted mice. Scale bars: 80 μm (original magnification, 10×). Lower panel demonstrates the entire transplanted lung under 2× magnification with control RAS lungs (B6D2F1/J → C57BL/6J), demonstrating thick pleural rind and fibrosis emanating along the bronchovascular bundle. B6D2F1/J → *AID*^–/–^ *μs*^–/–^ C57BL/6J allografts demonstrated substantial protection from fibrosis with preservation of lung ventilation.

**Figure 7:**
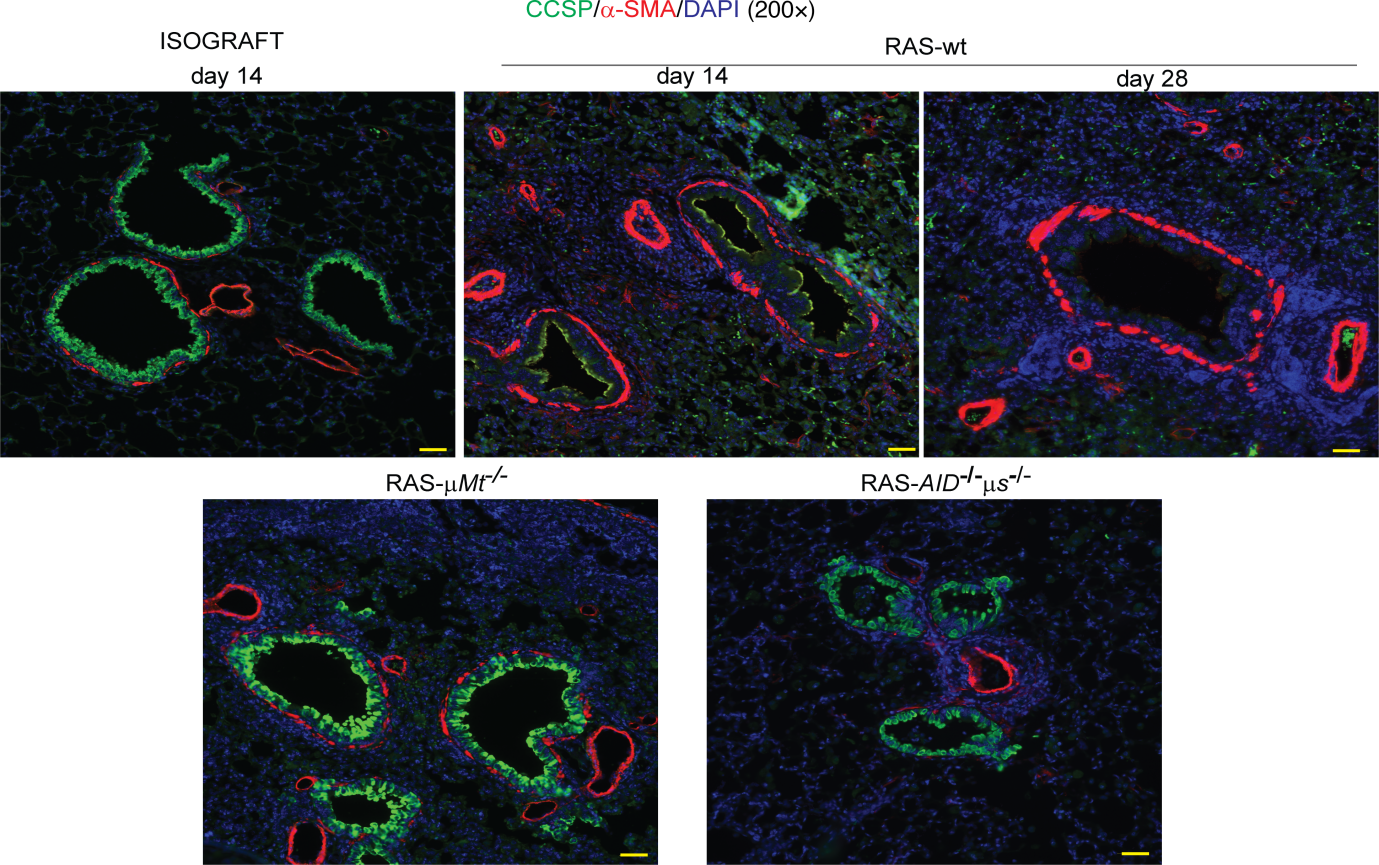
Epithelial mesenchymal tropic unit as a target of humoral immune responses in RAS. Dual immunofluorescent staining demonstrating loss of CCSP expression in bronchial epithelial cells in RAS allografts, along with expansion of α-SMA expressing mesenchymal cells in the subbronchial space. RAS allografts transplanted into *μMt^–/–^* and *AID*^–/–^ *μs*^–/–^ recipient mice demonstrated preservation of CCSP expressing bronchial epithelial cells. *n* = 3 in each group. Scale bars: 40 μm.

In addition to their role in antibody production and secretion, B cells can regulate immune responses through antigen presentation or cytokine production (22, 23). To further investigate whether B cells mediate the pathogenesis of RAS through secretion of antibodies, we used activation-induced cytidine deaminase (*AID*)/secretory μ-chain (*μs*) double-KO (*AID^−/−^μs^−/−^*) mice as allograft recipients. In these mice, B cells demonstrate a normal diverse repertoire of receptors but are unable to synthesize secretory immunoglobulins and exhibit a deficiency in plasma cells (24, 25). Notably, similar to our observations after transplantation of B6D2F1/J F1 allografts into *μMt^–/–^* B6 recipients, *AID^−/−^μs^−/−^* hosts did not develop the fibrosis that we observed in WT recipients (Figure 6, A and B). Also, the extent of lung injury was markedly decreased and fibrinous exudates were not detected (Figure 6B). Costaining for club cells with club cell secretory protein (CCSP) and myofibroblasts with α-smooth muscle actin (α-SMA) was performed in WT, *μMt^–/–^*, and *AID^–/–^μs^–/–^* recipients to investigate if disruption of the mesenchymal epithelial trophic unit is dependent upon B cells in the RAS model (Figure 7). Substantial loss of club cells was noted on days 14 and 28 in the RAS allografts (Figure 7). In stark contrast, however, CCSP expression was preserved in allografts that were placed in *μMt^–/–^* and *AID^–/–^μs^–/–^* recipients.

## Discussion

The primary cause of death after the first year of lung transplantation is chronic graft failure arising from fibrotic remodeling of the allograft subjected to repeated alloimmune and nonimmune insults (2). Small airways are a principal target of chronic allograft rejection, with BO being the most common histologic manifestation. However, a more robust form of fibrosis with involvement of the pleural, airway, and interstitial compartments is seen in RAS, a recently recognized phenotype of CLAD associated with particularly poor outcomes (5). Clinical studies have offered insight into physiological and histologic features of RAS, but pathogenic mechanisms leading to its development remain to be elucidated. In this study, by identifying an allogeneic mismatch combination in the murine orthotopic lung transplant model, which mimics histopathological changes of RAS, we demonstrate that humoral immune activation is critical in skewing the graft injury and remodeling responses toward a RAS phenotype. We provide the first evidence to our knowledge for a requisite role for B cells and secretory immunoglobulins in the development of RAS and offer key insights into the temporal evolution of allograft fibrogenesis — findings that have significant implications in clinical management of these patients.

A key finding of our study is the demonstration that humoral immune responses are requisite in the pathogenic evolution of RAS features. We have previously used F1 → parent mouse lung transplants to model BO and have demonstrated that B6D2F1/J donor lungs transplanted into parent DBA/2J mice demonstrate evolution from moderate lymphocytic infiltration to BO, with fibrosis and injury primarily confined to the bronchovascular bundles (19). The development of RAS features after transplanting the same F1 mouse into the other parent mouse (C57BL/6) pointed to differences in alloimmune responses between the 2 strain combinations. These findings have precedence in the fields of GVHD and autoimmune connective tissue diseases where transplantation of parent lymphocytes into F1 mice is used and different immune activation and disease phenotypes have been observed between F1 mice receiving the 2 parent cells (16–18). Global genome-wide comparison of the 2 models in our study revealed humoral immune response pathways as being differentially enriched in the RAS murine model, with significant upregulation of the genes in B cell activation pathways persisting over time in the RAS lungs. These findings were further substantiated by findings of marked B cell and plasma cell infiltration in RAS allografts and the presence of circulating DSA in the serum. Patients with persistent DSA are at a higher risk for developing CLAD and, more specifically, RAS (13, 14). RAS is the dominant form of allograft failure seen in patients with AMR (11), and explants from patients with RAS often demonstrate the presence of lymphoid follicles with B cells (11). While these studies have suggested a link between humoral immune responses and RAS, investigations of pathogenic mechanisms that drive this distinctive allograft pathology have been limited by the lack of a representative animal model (15). Our studies using this newly described model of RAS demonstrate a requisite role for humoral immunity in this aggressive form of CLAD presentation. Decreased allograft fibrogenesis was noted in RAS allografts transplanted into recipient lacking B cell (μMt^–/–^) or antibody secretion (AID^–/–^μs^–/–^) in our studies. Significant decreases in endothelialitis, fibrinous exudates, and macrophage infiltration were also seen in B cell–deficient mice, suggesting a role for humoral cell activation in the development of these histologic features. However, it is important to note that understanding of clinical CLAD phenotypes in human lung transplant recipients is still evolving, with underlying complex pathogenic mechanisms that cannot be fully emulated by murine models. Therefore, while our data support a more personalized approach to immunomodulation based on clinical and histopathologic characteristics, future work is needed to improve upon biomarkers of humoral immune activation and to decipher the overlap between immunological and clinical phenotypes.

We demonstrate that the B6D2F1/J → C57BL/6J model displays a spectrum of histopathological abnormalities noted in human RAS lungs, and we offer insights into the temporal evolution of this often fatal condition. The anatomic features of RAS are complex, with many different histologic patterns reported in human lungs that were either biopsied, explanted, or examined at autopsy (5, 8–10). Our ability to evaluate the lungs at various time points after transplant offered unique insights into the progression of these histopathological changes. Acute rejection with lymphocytic infiltration around blood vessels and airways has been commonly described in RAS lungs. We found that lymphocytic cellular rejection precedes and accompanies the development of other histological manifestations of RAS. Perivascular and peribronchial lymphocytic infiltrates were noted early, and significant infiltrates were still found at day 40. A unique pattern of lymphocytic aggregates in the bronchovascular bundles was identified in a significant number of allografts, similar to what has been described in human lungs with RAS. Acute fibrinous organizing pneumonia with fibrin exudates in the alveoli is a well-characterized pathologic feature of RAS (9). Fibrinous exudates were also a prominent feature in our model and were noted by day 14. They existed concomitantly with severe acute rejection, B lymphocyte and plasma cells infiltration, and endothelialitis but preceded influx of foamy macrophages, which are also a well-described feature in RAS lungs. At later time points, a higher degree of infiltration with macrophages correlated with decreased fibrinous exudates, suggesting a potential role for these macrophages in clearance of fibrin. RAS lungs have a heterogeneous appearance, and similar patterns were found in our murine lung allografts with circumscribed areas of fibrinous alveolar exudates, mostly centered around bronchovascular bundles, within areas of normal-appearing lung. Patchy ground glass opacities are a common early radiographic feature in patients with RAS and could be indicative of such a process, perhaps providing an opportunity to intervene before further evolution to fibrosis.

This newly described mouse model of RAS, in combination with our previously established model of BO (19), offers an opportunity for further mechanistic investigations into the pathogenesis of diverse CLAD endotypes. Clinically, CLAD ranges in spectrum from gradually progressive obstructive ventilatory defect arising from small airway limited fibrosis of BO, to a rapidly progressive restrictive decline pattern induced by robust pleural, interstitial, and bronchovascular bundle fibrosis of RAS (2, 26). However, the donor, recipient, and environmental factors that contribute to these diverse graft remodeling responses have remained elusive. The development of BO-like pathology in DBA/2J recipients versus RAS-like pathology in C57BL/6J recipients of the same F1 donor lung suggests a significant contribution of host factors in driving these diverse pathologies. Our present study focused on the humoral immune cell activation signature, which was found differentially in RAS versus BO allografts. However, further studies are needed to understand the mechanisms that drive this distinctive immune pathway activation in the C57BL/6J versus DBA/2J recipients. The contribution of specific differences between these species, such as decreased C5 protein in DBA/2J, need to be explored (27). Furthermore, the pathology of RAS with its distinctive features of fibrinous exudates, macrophage infiltration, and endothelial dysfunction overlaps with acute lung injury induced by diverse pulmonary pathogenic processes, including viral infections. Therefore, deciphering underlying pathogenic mechanisms in this model can also offer insight into lung injury and remodeling responses.

In summary, we describe a murine model of RAS after lung transplantation and demonstrate a critical role for humoral alloimmune responses in the pathogenesis of this phenotype. Our studies provide a window into the temporal evolution of this disease, information which can impact the care of these patients and aid in the development of biomarkers and diagnostic criteria. The unique pathogenic evolution of RAS and its dependence on B cells suggest the need for phenotype-specific therapeutic approaches.

## Methods

### Animals and orthotopic lung transplant model.

Specific pathogen–free male inbred mice B6D2F1/J (H2-b/d), C57BL/6J (H2-b), and *μMt^–/–^* mice were purchased from the Jackson Laboratory. *AID^–/–^μs^–/–^* mice were provided by Frances Lund and Troy Randall (University of Alabama, Birmingham, Alabama, USA) and Tasuku Honjo (Kyoto University, Kyoto, Japan). Both donors and recipients were mice aged between 8 and 12 weeks, and weighing 24–30 g. Isograft transplants were performed in the B6D2F1/J lungs → B6D2F1/J strain combination, and allogeneic transplants were performed in the B6D2F1/J lungs → C57BL/6J strain combination for the RAS model and B6D2F1/J lungs → DBA/2J for the BOS model. Orthotopic left lung transplantations were performed as previously described (19) using a surgical microscope (SZX16-SZX2; Olympus) with 2.1× to 34.5× magnifications for all procedures. Buprenorphine was given to recipient mice at the conclusion of the procedure and again every 12 hours until 3 days after transplant. No immunosuppressive drugs were used. Euthanasia protocols were approved by the University of Michigan IACUC were employed to sacrifice mice at 7, 14, 28, 40, and 60 days after transplantation.

### Histopathologic evaluation and IHC.

The heart and lung were removed en bloc, fixed in 10% formalin, and embedded in paraffin. Tissue sections of 5 μm thickness were stained with H&E to determine lung architecture and with Masson’s trichrome stain (NovaUltra Masson’s Trichrome Stain Kit; IHC World) in order to determine the presence of fibrosis in the lesions. Picrosirius red staining was performed as per manufacturer’s protocol (NovaUltra Sirius Red Stain Kits; IHC World). From each Picrosirius red–stained section, 4 random fields with bronchovascular bundles were imaged with an objective lens magnification at 10× and analyzed using ImageJ (ver. 1.52p; NIH) and a slightly modified protocol (28). To maintain uniform image size and scale, the scale was set as micrometers (Analyze → Set Scale). In order to isolate red-stained collagen, we changed the image type to RGB Stack that yields the gray-scale images of the channels (Image → Type → RGB Stack). In the Green channel, we set the threshold at 0–87 (Image → Adjust → Threshold). We recorded the area, area fraction, limit to threshold, and display label (Analyze → Measure). This morphometric assessment of collagen deposition was analyzed on GraphPad Prism (ver. 8.0.0). The pleural thickness was determined by measuring the distance between the pleural surface and the mesothelial basement membrane (29), in Masson’s trichrome–stained lung sections using ImageJ (ver. 1.52p) on 4 fields per slide (*n* = 4 representative sections per group). The micrograph of the entire lung was used to map some of these histological features.

Grading for histologic features was performed by pulmonary lung transplant pathologists on a scale of 0–3 in a blinded manner (30). The ratio of the average score across all samples to the highest score for the observed histologic feature was expressed as a heatmap (Figure 2C) with a fold change ranging from 0 to 1.0, wherein 1.0 represents severe phenotype. A categorical variable of presence of a histologic feature (defined as grade > 0) was used to determine percent incidence at each time point (Supplemental Table 1).

IHC staining was performed according to standard laboratory procedures using the following primary antibodies: rabbit anti-CD3 polyclonal antibody (1:500; Abcam), rabbit anti-B220 polyclonal antibody (1:400; Abcam), rabbit anti-CD138 (1:20; Thermo Fisher Scientific), and anti-GL7 (1:200; BioLegend), mouse anti–α-SMA (1:20; MilliporeSigma), and rabbit anti-CCSP (1:100; Abcam). Imaging was performed with an Olympus BX41 microscope connected to an Olympus DP20 camera.

### Collagen assay (hydroxyproline) and elastin ELISA.

Lung explants were homogenized in 1 mL of PBS; 1 mL of 12N HCl was added to the homogenate, and the samples were hydrolyzed at 120°C for 24 hours. A total of 5 μL of each sample was combined with 5 μL citrate/acetate buffer (238 mmol/L citric acid, 1.2% glacial acetic acid, 532 mmol/L sodium acetate, and 85 mmol/L sodium hydroxide) in a 96-well plate. A total of 100 μL of chloramine T solution (0.282 g chloramine T to 16 mL of citrate/acetate buffer, 2.0 mL of n-Propanol, and 2.0 mL double-distilled H_2_O) was then added for 30 minutes at room temperature followed by 100 μL of Ehrlich’s reagent (2.5 g paradimethylamino benzaldehyde added to 9.3 mL of n-Propanol and 3.9 mL of 70% perchloric acid), and incubation at 65°C for 30 minutes followed. The absorbance of each sample was measured at 550 nm. Standard curves for the experiment were generated using known concentrations of the hydroxyproline reagent (MilliporeSigma). RAS lung allografts were homogenized in PBS, and the homogenates were centrifuged at 10,621*g* for 10 minutes at 4°C. Supernatants were stored in –80°C until analyzed for elastin using a modified protocol (31).

### Flow cytometry and cell sorting.

Multichannel flow cytometric analysis was used to quantify inflammatory cell infiltration. Single-cell suspensions enriched for lung leukocytes were obtained from perfused and collagenase A–digested lungs and immunostained for 30 minutes with specific conjugated antibodies (BD biosciences) or isotype-matched controls at recommended concentrations. Stained cells were analyzed by flow cytometric analysis on a BD LR Fortessa (Becton Dickinson), and FlowJo software was used to calculate specific immune populations using established gating strategies. Initial gates were selected for CD45^+^ leukocytes, with subsequent gating to identify T cell populations that include CD3^+^, CD4^+^, and CD8^+^ T cells and to identify B cell populations that include CD19^+^ B cells, CD19^+^CD22^+^ activated B cells, CD19^+^CD138^+^ plasmablasts, and CD19^–^CD138^+^ plasma cells.

### Serum alloantibody titers.

Using a previously published protocol (21), 200 μL of PBS with 0.5% BSA and 0.02% sodium azide (PBA) containing 2 × 10^6^ thymocytes of donor origin (DBA/2J for isografts and RAS model; C57BL/6J for BOS model) were mixed with 200 μL of serially diluted serum for 1 hour at 4°C with frequent agitation. After 3 washes with PBA, cells were stained for 30 minutes at 4°C with 100 μL of PBA containing 1 μL of polyclonal fluorochrome–conjugated goat anti–mouse IgM (μ chain specific) or anti–mouse IgG (Fcγ fragment specific) (Jackson ImmunoResearch; catalog 115-116-075 and 115-095-071, respectively). Cells were analyzed on a FACScan (BD Biosciences), and FlowJo software (FlowJo) was used to calculate the median fluorescence intensity.

### Microarray analyses.

RAS allografts were homogenized, subjected to RNA isolation (74104, QIAGEN) and removal of DNA contamination (79254, QIAGEN). The RNA was then subjected to Affymetrix Microarray analyses using GeneTitan Mouse Gene 2.1 ST plate with the Affymetrix Plus reagent kit. RAS data and data previously collected from BOS models (unpublished, GeneTitan Mouse Gene 2.1 ST plate with the Affymetrix Plus reagent kit) were normalized using a robust multiarray (RMA) average for each gene (32). A weighted linear model designed for microarray analysis (33) was fit to the data to compute differential expression statistics between allograft and isograft data for each time point for the RAS and BOS transplant models, with year of data collection included in the model to account for batch effects. Samples were then weighted based on a gene-by-gene update algorithm designed to down-weight chips that are considered less reproducible (34). Probe sets were filtered to exclude probes with a variance of less than 0.05 and were limited to probe sets listed as “main” by Affymetrix. Differential expression was called using a fold-change threshold of > 2 or < –2 and a FDR-corrected *P* < 0.05 (35). The resulting differential expression data were uploaded to iPathway Guide (Advaita) for functional enrichment analysis (36). After *P* values were adjusted for multiple comparisons using FDR, tables of GO term (37, 38) enrichments for each time point were downloaded, including all terms (Supplemental Table 2) and limited to terms found to be enriched in RAS but not BOS (Supplemental Table 2). Volcano plots for RAS data were generated for GO:0042113, GO:0050853, GO:0002455, GO:0006958 using ggplot2 (ver. 3.2) (39) from tables of unfiltered log fold change (log_FC_) and FDR-adjusted *P* values for all genes annotated for that GO term at each time point. All analysis and graphics were generated in R (ver. 3.4.0 or ver. 3.6.1) unless otherwise indicated. Statistical comparisons of the distributions of log_FC_ between a pair of time points was compared using paired, 2-tailed *t* test.

### Accession numbers.

Microarray data were deposited in the Gene Expression Omnibus (GEO; https://www.ncbi.nlm.nih.gov/geo/), with accession no. GSE158057 for data presented in Figure 3.

### Statistics.

The Student’s 2-tailed *t* test was used to determine *P* values when comparing 2 groups. When comparing 3 or more groups, 1-way ANOVA was performed with a post hoc Bonferroni test to determine which groups showed significant differences, unless otherwise specified. *P* < 0.05 was considered significant using GraphPad Prism (ver. 8.0.0) for Windows 64-bit.

### Study approval.

All experiments were performed according to protocols approved by the University of Michigan IACUC.

## Author contributions

Conceptualization and experimental design were contributed by YA, NMW, KM, and VNL. Data acquisition, analysis, and interpretation were contributed by NMW, YA, KM, RRB, AL, DSW, RV, RH, WL, DK, JM, CFF, and VNL. Drafting of the manuscript was contributed by KM, RV, MPC, DK, and VNL.

## Supplementary Material

Supplemental data

## Figures and Tables

**Table 2:**
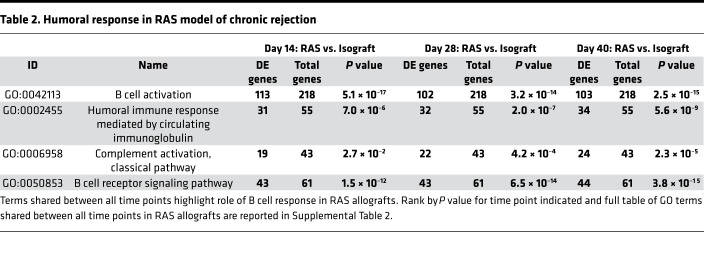
Humoral response in RAS model of chronic rejection

**Table 1:**
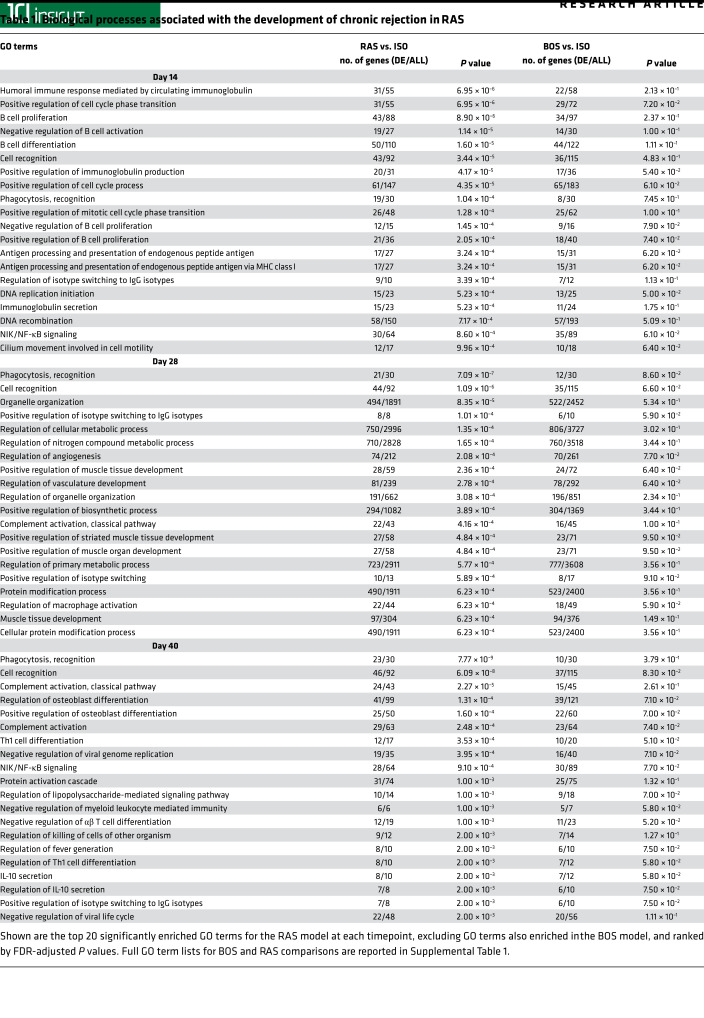
Biological processes associated with the development of chronic rejection in RAS

## Version 1 12/03/2020

Electronic publication
