# Supplementary material for: Humoral immune responses mediate the development of a restrictive phenotype of chronic lung allograft dysfunction
Source: JCI Insight. 2020 Dec 3;5(23):e136533. doi: 10.1172/jci.insight.136533 (PMC7714414; doi:10.1172/jci.insight.136533)
Supplement: Supplemental data [file jciinsight-5-136533-s111.pdf]

Humoral Immune Responses Mediate the Development of a Restrictive Phenotype of Chronic Lung Allograft Dysfunction

Keizo Misumi[1], David S. Wheeler[1], Yoshiro Aoki[1], Michael P. Combs[1], Russell R. Braeuer[1], Ryuji Higashikubo[2], Wenjun Li[2], Daniel Kreisel[2], Ragini Vittal[1], Jeffrey Myers[3], Amir Lagstein[3], Natalie M. Walker[1], Carol F. Farver[3] and Vibha N. Lama[1]

[1] Division of Pulmonary and Critical Care Medicine, Department of Internal Medicine, University of Michigan, Ann Arbor, MI48109

[2] Department of Surgery, Washington University in St. Louis, St. Louis, MO63130

[3] Department of Pathology, University of Michigan, Ann Arbor, MI48109

Running Title: Humoral Immunity in RAS

Corresponding Address:

Vibha N. Lama, M.D., M.S.

Henry Sewall Research Professor of Pulmonary and Critical Care Medicine

Professor of Internal Medicine

Associate Chief, Division of Pulmonary & Critical Care Medicine

University of Michigan Health System

1150 W. Medical Center Drive, 6301 MSRB III Ann Arbor, MI 48109-0644

Phone: 734-936-5047; Fax: 734-936-5048

vlama@umich.edu

Supplemental Figure 1

RAS allograft day 28

Artery with Endothelialitis

Vein with Endothelialitis

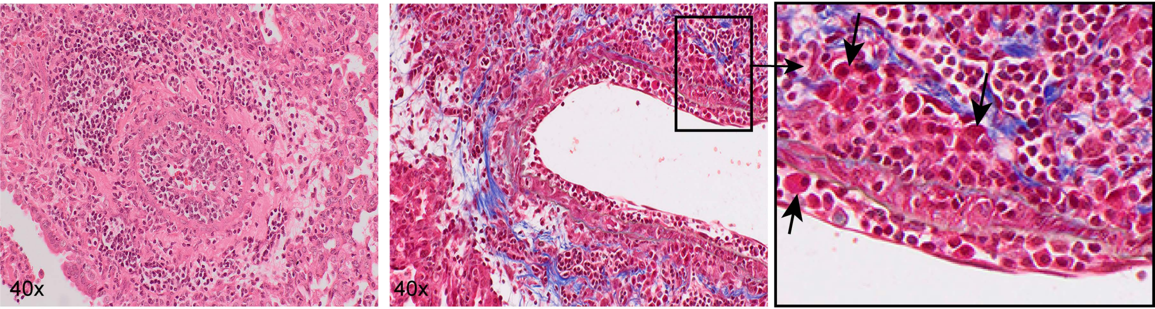

Vein with Endothelialitis

RAS-wt

RAS-μ*Mt-/-*

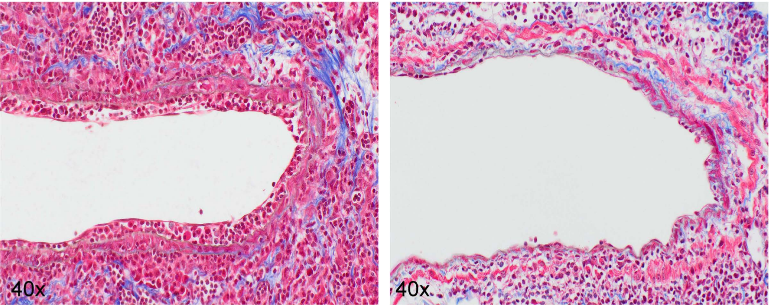

Supplemental Figure 2

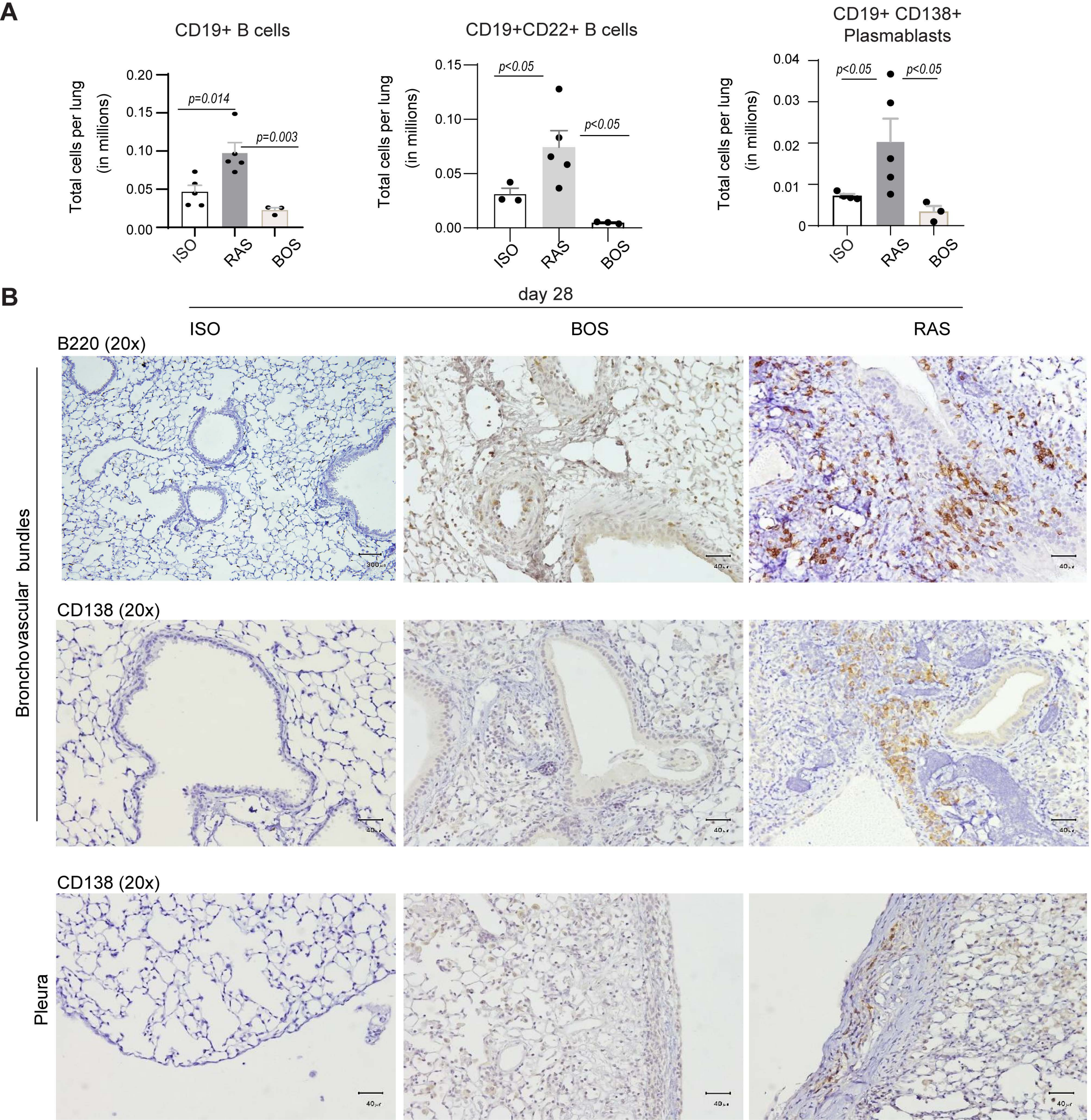

Supplemental Table 1. Percent incidence of histological features at various time points in the RAS allografts.

Days post-transplant	7	14	28	40	60
Acute rejection: A	100%	100%	100%	100%	100%
Acute rejection: B	100%	100%	100%	100%	100%
Pleuritis	100%	100%	100%	100%	100%
Pleural fibrosis (PF)	17%	100%	100%	100%	100%
Pleuritis with plasma cells	0%	60%	90%	100%	100%
Broncho vascular bundle fibrosis (BVBF)	0%	100%	100%	100%	100%
Fibrous exudates (FE)	0%	100%	70%	88%	33%
Plasmacytic vasculitis	0%	0%	70%	88%	100%
Endothelialitis	0%	0%	60%	100%	67%
Foamy macrophages	0%	0%	80%	88%	67%
PPFE	0%	0%	0%	25%	33%

The ratio of the number of mice that presented the specific histopathological features in Figure 2C at each timepoint to the total number of mice in the group.

Supplemental Table 2 Key. Comparisons of the full GO term lists for BOS and RAS allografts.

GO term enrichment results for Biological Processes downloaded from iPathway Guide report (Advaita: https://ipathwayguide.advaitabio.com)

Column Key:

GO_Id	=	GO term ID number
goName	=	GO term name
countDE	=	DE gene count in GO term for comparison
countAll	=	Total measured gene count for comparison in GO term
pv_fdr	=	FDR corrected p-value for overrepresentation analysis

Table descriptions

RAS-BOS Day 14 GO terms = GO terms enriched in RAS (B6D2F1/J -> C57BL/6J), 14 hour timepoint, excluding GO terms also enriched in BOS (B6D2F1/J → DBA2J), 14 hour timepoint; iPathway Guide enrichment analysis

RAS-BOS Day 28 GO terms = GO terms enriched in RAS, 28 hour timepoint, excluding GO terms also enriched in BOS, 28 hour timepoint; iPathway Guide enrichment analysis

RAS-BOS Day 40 terms = GO terms enriched in RAS, 40 hour timepoint, excluding GO terms also enriched in BOS, 40 hour timepoint; iPathway Guide enrichment analysis

RAS_BOS Day 14 GO Terms

GO_Id	goName	RAS_Day14_countDE	RAS_Day14_countAll	RAS_Day14_pv_fdr	BOS_Day14_countDE	BOS_Day14_countAll	BOS_Day14_pv_fdr
GO:0002455	humoral immune response mediated by circulating immunoglobulin	31	55	6.94933E-06	22	58	0.212651549
GO:1901989	positive regulation of cell cycle phase transition	31	55	6.94933E-06	29	72	0.072230941
GO:0042100	B cell proliferation	43	88	8.90139E-06	34	97	0.236991882
GO:0050869	negative regulation of B cell activation	19	27	1.13799E-05	14	30	0.099512844
GO:0030183	B cell differentiation	50	110	1.60369E-05	44	122	0.110664582
GO:0008037	cell recognition	43	92	3.44093E-05	36	115	0.482610776
GO:0002639	positive regulation of immunoglobulin production	20	31	0.000041696	17	36	0.054228158
GO:0090068	positive regulation of cell cycle process	61	147	4.35157E-05	65	183	0.061287984
GO:0006910	phagocytosis, recognition	19	30	0.000104057	8	30	0.744616472
GO:1901992	positive regulation of mitotic cell cycle phase transition	26	48	0.000128046	25	62	0.100421917
GO:0030889	negative regulation of B cell proliferation	12	15	0.000145369	9	16	0.079396904
GO:0030890	positive regulation of B cell proliferation	21	36	0.000205062	18	40	0.07394458
GO:0002483	antigen processing and presentation of endogenous peptide antigen	17	27	0.000323727	15	31	0.061548653
GO:0019885	antigen processing and presentation of endogenous peptide antigen via	17	27	0.000323727	15	31	0.061548653
GO:0048302	regulation of isotype switching to IgG isotypes	9	10	0.000338861	7	12	0.112955303
GO:0006270	DNA replication initiation	15	23	0.000522737	13	25	0.050254722
GO:0048305	immunoglobulin secretion	15	23	0.000522737	11	24	0.174656784
GO:0006310	DNA recombination	58	150	0.000716837	57	193	0.509093514
GO:0038061	NIK/NF-kappaB signaling	30	64	0.000860017	35	89	0.060591654
GO:0060294	cilium movement involved in cell motility	12	17	0.000995551	10	18	0.064486504
GO:0000018	regulation of DNA recombination	26	53	0.001007361	25	69	0.244026541
GO:0045622	regulation of T-helper cell differentiation	17	29	0.001071356	16	33	0.050437366
GO:0000727	double-strand break repair via break-induced replication	8	9	0.001167834	7	11	0.07394458
GO:0002579	positive regulation of antigen processing and presentation	8	9	0.001167834	7	11	0.07394458
GO:0042535	positive regulation of tumor necrosis factor biosynthetic process	10	13	0.001286914	9	15	0.050759043
GO:0002484	antigen processing and presentation of endogenous peptide antigen via	14	22	0.001313593	13	26	0.06995675
GO:0002476	antigen processing and presentation of endogenous peptide antigen via	14	22	0.001313593	13	26	0.06995675
GO:0033631	cell-cell adhesion mediated by integrin	9	11	0.001313593	8	15	0.136929801
GO:0048291	isotype switching to IgG isotypes	9	11	0.001313593	7	13	0.170890874
GO:0002200	somatic diversification of immune receptors	21	40	0.001374902	18	51	0.377564799
GO:0030317	flagellated sperm motility	31	69	0.001658992	30	73	0.050584003
GO:0045730	respiratory burst	13	20	0.001658992	11	24	0.174656784
GO:0097722	sperm motility	31	69	0.001658992	30	73	0.050584003
GO:0033044	regulation of chromosome organization	55	145	0.001910157	71	218	0.215755529
GO:0042462	eye photoreceptor cell development	12	18	0.002171667	9	26	0.496013343
GO:0090022	regulation of neutrophil chemotaxis	16	28	0.002421058	12	30	0.287555049
GO:0002428	antigen processing and presentation of peptide antigen via MHC class Ib	14	23	0.002542439	13	27	0.093710429
GO:1902622	regulation of neutrophil migration	19	36	0.002542439	17	37	0.070984826
GO:0001923	B-1 B cell differentiation	6	6	0.002635651	4	6	0.207815495
GO:0050859	negative regulation of B cell receptor signaling pathway	6	6	0.002635651	4	6	0.207815495
GO:0002664	regulation of T cell tolerance induction	6	6	0.002635651	6	9	0.087106314
GO:0030168	platelet activation	27	59	0.002881005	25	64	0.134153197
GO:2001251	negative regulation of chromosome organization	28	62	0.003000651	35	92	0.093723268
GO:0002486	antigen processing and presentation of endogenous peptide antigen via	13	21	0.003205412	12	25	0.109151612
GO:0043373	CD4-positive, alpha-beta T cell lineage commitment	10	14	0.003205412	9	15	0.050759043
GO:0031649	heat generation	13	21	0.003205412	11	20	0.052209345
GO:0036158	outer dynein arm assembly	10	14	0.003205412	9	15	0.050759043
GO:0050855	regulation of B cell receptor signaling pathway	10	14	0.003205412	7	15	0.278976678
GO:0032647	regulation of interferon-alpha production	13	21	0.003205412	12	23	0.061501911
GO:0044346	fibroblast apoptotic process	9	12	0.003686537	9	18	0.154912533
GO:0048304	positive regulation of isotype switching to IgG isotypes	7	8	0.003785956	5	10	0.324790117
GO:0002604	regulation of dendritic cell antigen processing and presentation	7	8	0.003785956	5	9	0.244026541
GO:0060700	regulation of ribonuclease activity	7	8	0.003785956	7	11	0.07394458
GO:0002517	T cell tolerance induction	7	8	0.003785956	7	11	0.07394458
GO:0006302	double-strand break repair	44	113	0.003883942	49	152	0.326918109
GO:0000724	double-strand break repair via homologous recombination	31	72	0.003883942	32	91	0.240940767
GO:0000725	recombinational repair	31	72	0.003883942	32	91	0.240940767
GO:0002468	dendritic cell antigen processing and presentation	8	10	0.003980525	6	11	0.20919096
GO:1900165	negative regulation of interleukin-6 secretion	8	10	0.003980525	6	10	0.139863469
GO:0042461	photoreceptor cell development	16	29	0.003980525	10	40	0.815748041
GO:0045625	regulation of T-helper 1 cell differentiation	8	10	0.003980525	7	12	0.112955303
GO:0002827	positive regulation of T-helper 1 type immune response	12	19	0.004189314	11	23	0.135313214
GO:0046849	bone remodeling	33	79	0.004732666	33	83	0.061053285
GO:0006959	humoral immune response	61	171	0.005128513	59	183	0.278489136
GO:0061512	protein localization to cilium	18	35	0.005128513	16	38	0.160548564
GO:1901222	regulation of NIK/NF-kappaB signaling	27	61	0.005128513	33	85	0.082822243
GO:0016444	somatic cell DNA recombination	18	35	0.005128513	16	47	0.471158597
GO:0002562	somatic diversification of immune receptors via germline recombination	18	35	0.005128513	16	47	0.471158597
GO:0043369	CD4-positive or CD8-positive, alpha-beta T cell lineage commitment	11	17	0.005217929	10	19	0.093723268
GO:0045063	T-helper 1 cell differentiation	11	17	0.005217929	9	20	0.244026541
GO:0045880	positive regulation of smoothened signaling pathway	16	30	0.006186697	16	33	0.050437366
GO:0071622	regulation of granulocyte chemotaxis	21	44	0.006289341	17	45	0.274658547
GO:0002363	alpha-beta T cell lineage commitment	10	15	0.006643868	9	16	0.079396904
GO:2000116	regulation of cysteine-type endopeptidase activity	54	149	0.006643868	63	179	0.078430159
GO:1905521	regulation of macrophage migration	17	33	0.006842888	16	35	0.085950985
GO:0016445	somatic diversification of immunoglobulins	17	33	0.006842888	15	42	0.408188163
GO:0030522	intracellular receptor signaling pathway	42	110	0.007663049	43	144	0.510682797
GO:0045190	isotype switching	15	28	0.008206128	14	36	0.287079542
GO:0045830	positive regulation of isotype switching	9	13	0.008206128	7	17	0.414817346
GO:0002208	somatic diversification of immunoglobulins involved in immune respons	15	28	0.008206128	14	36	0.287079542
GO:0002204	somatic recombination of immunoglobulin genes involved in immune re	15	28	0.008206128	14	36	0.287079542
GO:0002295	T-helper cell lineage commitment	9	13	0.008206128	8	14	0.097253631
GO:2000351	regulation of endothelial cell apoptotic process	20	42	0.008401103	20	46	0.079168355
GO:0060287	epithelial cilium movement involved in determination of left/right asym	5	5	0.00886916	4	6	0.207815495
GO:0031536	positive regulation of exit from mitosis	5	5	0.00886916	4	6	0.207815495
GO:0060558	regulation of calcidiol 1-monooxygenase activity	5	5	0.00886916	3	6	0.482610776
GO:0042430	indole-containing compound metabolic process	11	18	0.009613845	9	18	0.154912533
GO:0033630	positive regulation of cell adhesion mediated by integrin	11	18	0.009613845	10	19	0.093723268
GO:0045624	positive regulation of T-helper cell differentiation	11	18	0.009613845	11	21	0.075976733
GO:0001771	immunological synapse formation	8	11	0.009739055	7	15	0.278976678
GO:0090594	inflammatory response to wounding	8	11	0.009739055	7	13	0.170890874
GO:2000269	regulation of fibroblast apoptotic process	8	11	0.009739055	8	17	0.243761743
GO:0072540	T-helper 17 cell lineage commitment	8	11	0.009739055	7	12	0.112955303
GO:0044839	cell cycle G2/M phase transition	31	76	0.010008744	32	88	0.179082699
GO:0042073	intraciliary transport	17	34	0.010008744	14	39	0.414817346
GO:0016032	viral process	51	142	0.010849469	64	190	0.153401114
GO:0006586	indolalkylamine metabolic process	7	9	0.011183644	5	9	0.244026541
GO:0072610	interleukin-12 secretion	7	9	0.011183644	5	9	0.244026541
GO:0006568	tryptophan metabolic process	7	9	0.011183644	5	9	0.244026541

GO:0002634	regulation of germinal center formation	6	7	0.011373534	5	8	0.171041956
GO:0034103	regulation of tissue remodeling	26	61	0.011373534	26	69	0.175283897
GO:0060556	regulation of vitamin D biosynthetic process	6	7	0.011373534	4	8	0.402682328
GO:0046549	retinal cone cell development	6	7	0.011373534	5	8	0.171041956
GO:0002246	wound healing involved in inflammatory response	6	7	0.011373534	6	9	0.087106314
GO:1902749	regulation of cell cycle G2/M phase transition	25	58	0.011651577	21	69	0.55212829
GO:1904019	epithelial cell apoptotic process	32	80	0.011995873	35	89	0.060591654
GO:0042226	interleukin-6 biosynthetic process	12	21	0.012185226	11	25	0.220157552
GO:0032692	negative regulation of interleukin-1 production	15	29	0.012185226	12	30	0.287555049
GO:0045911	positive regulation of DNA recombination	12	21	0.012185226	8	28	0.687331495
GO:0045408	regulation of interleukin-6 biosynthetic process	12	21	0.012185226	11	24	0.174656784
GO:0016447	somatic recombination of immunoglobulin gene segments	15	29	0.012185226	14	39	0.414817346
GO:0002360	T cell lineage commitment	12	21	0.012185226	11	23	0.135313214
GO:1990774	tumor necrosis factor secretion	15	29	0.012185226	12	31	0.341410286
GO:0007599	hemostasis	43	116	0.012275564	43	124	0.193480303
GO:0001780	neutrophil homeostasis	10	16	0.012329462	10	18	0.064486504
GO:0032727	positive regulation of interferon-alpha production	10	16	0.012329462	8	18	0.287555049
GO:0019079	viral genome replication	24	56	0.015335308	29	71	0.060916147
GO:1901224	positive regulation of NIK/NF-kappaB signaling	19	41	0.015376398	24	62	0.15808473
GO:0042531	positive regulation of tyrosine phosphorylation of STAT protein	19	41	0.015376398	20	45	0.063156298
GO:0010389	regulation of G2/M transition of mitotic cell cycle	22	50	0.015645966	20	57	0.361468407
GO:0031664	regulation of lipopolysaccharide-mediated signaling pathway	9	14	0.01605966	9	18	0.154912533
GO:0046639	negative regulation of alpha-beta T cell differentiation	11	19	0.016324377	11	23	0.135313214
GO:0045191	regulation of isotype switching	11	19	0.016324377	11	25	0.220157552
GO:0006281	DNA repair	73	222	0.017470936	95	306	0.285072331
GO:0060249	anatomical structure homeostasis	78	240	0.017875266	94	283	0.098114007
GO:0007596	blood coagulation	42	115	0.018292813	42	123	0.236382831
GO:0071356	cellular response to tumor necrosis factor	41	112	0.019353382	49	132	0.058856175
GO:0032691	negative regulation of interleukin-1 beta production	12	22	0.019604771	9	22	0.34901769
GO:0048143	astrocyte activation	8	12	0.020431797	9	16	0.079396904
GO:0043374	CD8-positive, alpha-beta T cell differentiation	8	12	0.020431797	8	15	0.136929801
GO:0045410	positive regulation of interleukin-6 biosynthetic process	8	12	0.020431797	8	14	0.097253631
GO:0034162	toll-like receptor 9 signaling pathway	8	12	0.020431797	7	13	0.170890874
GO:0045069	regulation of viral genome replication	21	48	0.020507779	25	61	0.084949787
GO:0046530	photoreceptor cell differentiation	19	42	0.020583623	16	54	0.640829083
GO:0002313	mature B cell differentiation involved in immune response	10	17	0.021693189	10	18	0.064486504
GO:0044458	motile cilium assembly	13	25	0.022136989	11	22	0.102389696
GO:0043032	positive regulation of macrophage activation	13	25	0.022136989	12	27	0.182024141
GO:0060142	regulation of syncytium formation by plasma membrane fusion	13	25	0.022136989	13	25	0.050254722
GO:0046885	regulation of hormone biosynthetic process	14	28	0.024233256	14	31	0.122322265
GO:1904467	regulation of tumor necrosis factor secretion	14	28	0.024233256	11	29	0.398341123
GO:0050817	coagulation	42	117	0.025345712	42	125	0.250530856
GO:0098586	cellular response to virus	15	31	0.025602807	13	35	0.378627816
GO:0038094	Fc-gamma receptor signaling pathway	7	10	0.025602807	7	11	0.07394458
GO:0001781	neutrophil apoptotic process	7	10	0.025602807	7	11	0.07394458
GO:0033260	nuclear DNA replication	15	31	0.025602807	14	29	0.075976733
GO:0031620	regulation of fever generation	7	10	0.025602807	6	10	0.139863469
GO:1902751	positive regulation of cell cycle G2/M phase transition	11	20	0.026195965	6	24	0.809025373
GO:0043243	positive regulation of protein complex disassembly	11	20	0.026195965	10	22	0.212651549
GO:0006471	protein ADP-ribosylation	11	20	0.026195965	11	23	0.135313214
GO:0048538	thymus development	16	34	0.026535465	18	42	0.109151612
GO:0070507	regulation of microtubule cytoskeleton organization	41	114	0.02664714	53	147	0.078133704
GO:0006958	complement activation, classical pathway	19	43	0.027275233	11	45	0.826077288
GO:0043371	negative regulation of CD4-positive, alpha-beta T cell differentiation	9	15	0.028721923	8	20	0.414743197
GO:0070269	pyroptosis	9	15	0.028721923	8	17	0.243761743
GO:0032069	regulation of nuclease activity	9	15	0.028721923	11	20	0.052209345
GO:0033326	cerebrospinal fluid secretion	4	4	0.029607664	3	4	0.240940767
GO:2000270	negative regulation of fibroblast apoptotic process	4	4	0.029607664	4	7	0.287555049
GO:0043569	negative regulation of insulin-like growth factor receptor signaling pathv	4	4	0.029607664	3	6	0.482610776
GO:0002636	positive regulation of germinal center formation	4	4	0.029607664	3	4	0.240940767
GO:0090267	positive regulation of mitotic cell cycle spindle assembly checkpoint	4	4	0.029607664	3	5	0.361468407
GO:0090023	positive regulation of neutrophil chemotaxis	12	23	0.029607664	8	24	0.547549482
GO:0043552	positive regulation of phosphatidylinositol 3-kinase activity	12	23	0.029607664	12	26	0.142893975
GO:0090232	positive regulation of spindle checkpoint	4	4	0.029607664	3	5	0.361468407
GO:0002666	positive regulation of T cell tolerance induction	4	4	0.029607664	4	5	0.106897855
GO:1901894	regulation of calcium-transporting ATPase activity	4	4	0.029607664	2	6	0.687331495
GO:0051136	regulation of NK T cell differentiation	4	4	0.029607664	4	5	0.106897855
GO:2001187	positive regulation of CD8-positive, alpha-beta T cell activation	6	8	0.030142733	6	9	0.087106314
GO:0033632	regulation of cell-cell adhesion mediated by integrin	6	8	0.030142733	6	11	0.20919096
GO:0090266	regulation of mitotic cell cycle spindle assembly checkpoint	6	8	0.030142733	5	10	0.324790117
GO:1903504	regulation of mitotic spindle checkpoint	6	8	0.030142733	5	10	0.324790117
GO:0090231	regulation of spindle checkpoint	6	8	0.030142733	5	10	0.324790117
GO:0017085	response to insecticide	6	8	0.030142733	3	9	0.641639495
GO:0042670	retinal cone cell differentiation	6	8	0.030142733	6	9	0.087106314
GO:0042368	vitamin D biosynthetic process	6	8	0.030142733	4	10	0.507427556
GO:0010543	regulation of platelet activation	13	26	0.032067197	13	28	0.115670109
GO:0046218	indolalkylamine catabolic process	5	6	0.033055831	4	7	0.287555049
GO:0042436	indole-containing compound catabolic process	5	6	0.033055831	4	7	0.287555049
GO:0060335	positive regulation of interferon-gamma-mediated signaling pathway	5	6	0.033055831	5	7	0.100421917
GO:0031666	positive regulation of lipopolysaccharide-mediated signaling pathway	5	6	0.033055831	5	8	0.171041956
GO:0060332	positive regulation of response to interferon-gamma	5	6	0.033055831	5	7	0.100421917
GO:0014074	response to purine-containing compound	27	69	0.033055831	30	89	0.340876171
GO:0002291	T cell activation via T cell receptor contact with antigen bound to MHC n	5	6	0.033055831	5	7	0.100421917
GO:0006569	tryptophan catabolic process	5	6	0.033055831	4	7	0.287555049
GO:1902624	positive regulation of neutrophil migration	14	29	0.033286015	11	29	0.398341123
GO:0032890	regulation of organic acid transport	21	50	0.033286015	21	59	0.32008596
GO:0022617	extracellular matrix disassembly	10	18	0.034133162	11	23	0.135313214
GO:0032957	inositol trisphosphate metabolic process	10	18	0.034133162	12	23	0.061501911
GO:0010971	positive regulation of G2/M transition of mitotic cell cycle	10	18	0.034133162	6	21	0.704132645
GO:0051023	regulation of immunoglobulin secretion	10	18	0.034133162	8	19	0.34901769
GO:0006066	alcohol metabolic process	60	182	0.034189383	81	233	0.052009004
GO:0070542	response to fatty acid	15	32	0.034329038	16	40	0.227179564
GO:0070527	platelet aggregation	16	35	0.034940782	16	39	0.192919372
GO:0006956	complement activation	25	63	0.035987619	19	64	0.619887123
GO:0050711	negative regulation of interleukin-1 secretion	8	13	0.036357078	6	13	0.341986498
GO:0045623	negative regulation of T-helper cell differentiation	8	13	0.036357078	7	17	0.414817346
GO:1903975	regulation of glial cell migration	8	13	0.036357078	8	16	0.191409839
GO:0009725	response to hormone	124	417	0.036357078	162	509	0.085156339
GO:0006811	ion transport	270	977	0.037228571	343	1131	0.0818275
GO:0055003	cardiac myofibril assembly	11	21	0.038794558	11	23	0.135313214
GO:0042104	positive regulation of activated T cell proliferation	11	21	0.038794558	10	24	0.287555049

GO:0060143	positive regulation of syncytium formation by plasma membrane fusion	11	21	0.038794558	11	21	0.075976733
GO:0050792	regulation of viral process	34	93	0.038927125	41	121	0.244026541
GO:0051054	positive regulation of DNA metabolic process	46	134	0.039110328	57	165	0.12647925
GO:0035051	cardiocyte differentiation	43	124	0.041324003	52	147	0.106694753
GO:0046165	alcohol biosynthetic process	33	90	0.041372775	41	112	0.106796695
GO:0008630	intrinsic apoptotic signaling pathway in response to DNA damage	26	67	0.042098471	33	87	0.106897855
GO:1904646	cellular response to amyloid-beta	12	24	0.042161787	13	29	0.150033256
GO:0046850	regulation of bone remodeling	20	48	0.043299692	21	49	0.080879129
GO:1903900	regulation of viral life cycle	29	77	0.043586902	34	96	0.212651549
GO:0043281	regulation of cysteine-type endopeptidase activity involved in apoptotic	44	128	0.044457642	57	160	0.080221956
GO:0019674	NAD metabolic process	13	27	0.044515019	10	35	0.679023173
GO:0010758	regulation of macrophage chemotaxis	13	27	0.044515019	12	27	0.182024141
GO:0019058	viral life cycle	37	104	0.044719756	47	130	0.098121772
GO:0022411	cellular component disassembly	61	188	0.04492418	80	244	0.164816165
GO:0034104	negative regulation of tissue remodeling	9	16	0.045808439	7	20	0.510682797
GO:0002726	positive regulation of T cell cytokine production	9	16	0.045808439	10	19	0.093723268
GO:0001754	eye photoreceptor cell differentiation	14	30	0.045897308	13	37	0.462817974
GO:0072376	protein activation cascade	28	74	0.045897308	22	75	0.632071278
GO:0098656	anion transmembrane transport	36	101	0.047559013	34	131	0.812030238
GO:0035721	intraciliary retrograde transport	7	11	0.047559013	8	13	0.061501911
GO:2000647	negative regulation of stem cell proliferation	7	11	0.047559013	5	15	0.625786591
GO:0044546	NLRP3 inflammasome complex assembly	7	11	0.047559013	7	13	0.170890874
GO:0051984	positive regulation of chromosome segregation	7	11	0.047559013	7	20	0.510682797
GO:0045815	positive regulation of gene expression, epigenetic	7	11	0.047559013	7	25	0.718396862
GO:0010919	regulation of inositol phosphate biosynthetic process	7	11	0.047559013	7	12	0.112955303
GO:0090036	regulation of protein kinase C signaling	7	11	0.047559013	9	15	0.050759043
GO:0032495	response to muramyl dipeptide	7	11	0.047559013	8	14	0.097253631
GO:0032494	response to peptidoglycan	7	11	0.047559013	7	12	0.112955303
GO:0034134	toll-like receptor 2 signaling pathway	7	11	0.047559013	6	14	0.414817346
GO:0009628	response to abiotic stimulus	178	626	0.047601375	228	724	0.050437366
GO:0006606	protein import into nucleus	27	71	0.047961283	32	92	0.244026541
GO:0043269	regulation of ion transport	144	497	0.049907908	176	549	0.054748292

RAS_BOS Day 28 GO Terms

GO_Id	goName	RAS_Day28_countDE	RAS_Day28_countAll	RAS_Day28_pv_fdr	BOS_Day28_countDE	BOS_Day28_countAll	BOS_Day28_pv_fdr
GO:0006910	phagocytosis, recognition	21	30	7.09E-07	12	30	0.085942646
GO:0008037	cell recognition	44	92	1.09221E-06	35	115	0.06627
GO:0006996	organelle organization	494	1891	8.347E-05	522	2452	0.534084626
GO:0048304	positive regulation of isotype switching to IgG isotypes	8	8	0.000101069	6	10	0.058959971
GO:0031323	regulation of cellular metabolic process	750	2996	0.000134895	806	3727	0.301565136
GO:0051171	regulation of nitrogen compound metabolic process	710	2828	0.000164685	760	3518	0.343578254
GO:0045765	regulation of angiogenesis	74	212	0.000208368	70	261	0.077202304
GO:1901863	positive regulation of muscle tissue development	28	59	0.000236337	24	72	0.063747488
GO:1901342	regulation of vasculature development	81	239	0.000278018	78	292	0.063747488
GO:0033043	regulation of organelle organization	191	662	0.000308442	196	851	0.234432208
GO:0009891	positive regulation of biosynthetic process	294	1082	0.000388628	304	1369	0.343598605
GO:0006958	complement activation, classical pathway	22	43	0.000415476	16	45	0.100054309
GO:0048636	positive regulation of muscle organ development	27	58	0.000484032	23	71	0.094792771
GO:0045844	positive regulation of striated muscle tissue development	27	58	0.000484032	23	71	0.094792771
GO:0080090	regulation of primary metabolic process	723	2911	0.000576747	777	3608	0.35647213
GO:0045830	positive regulation of isotype switching	10	13	0.000588668	8	17	0.090802077
GO:0006464	cellular protein modification process	490	1911	0.000622967	523	2400	0.35647213
GO:0060537	muscle tissue development	97	304	0.000622967	94	376	0.148835411
GO:0036211	protein modification process	490	1911	0.000622967	523	2400	0.35647213
GO:0043030	regulation of macrophage activation	22	44	0.000622967	18	49	0.058959971
GO:0045669	positive regulation of osteoblast differentiation	24	50	0.000666638	18	60	0.246170094
GO:0044085	cellular component biogenesis	434	1676	0.000692527	479	2130	0.124875
GO:0031328	positive regulation of cellular biosynthetic process	285	1057	0.00088466	295	1340	0.411871622
GO:0019222	regulation of metabolic process	808	3292	0.00088466	883	4072	0.229011929
GO:0010557	positive regulation of macromolecule biosynthetic process	269	993	0.000993881	273	1265	0.504629449
GO:0034104	negative regulation of tissue remodeling	11	16	0.001172643	8	20	0.190429181
GO:0038061	NIK/NF-kappaB signaling	28	64	0.00123443	29	89	0.050134527
GO:0010638	positive regulation of organelle organization	95	302	0.001272593	90	409	0.545618213
GO:0050679	positive regulation of epithelial cell proliferation	54	152	0.001432107	49	175	0.090802077
GO:1990774	tumor necrosis factor secretion	16	29	0.001432107	12	31	0.10699485
GO:0050859	negative regulation of B cell receptor signaling pathway	6	6	0.001527191	4	6	0.117133853
GO:0065003	protein-containing complex assembly	250	921	0.001527191	279	1197	0.095824007
GO:0048738	cardiac muscle tissue development	60	175	0.001738319	51	219	0.443727768
GO:0045191	regulation of isotype switching	12	19	0.001738319	11	25	0.058959971
GO:0060255	regulation of macromolecule metabolic process	741	3018	0.001848972	799	3740	0.443727768
GO:0031620	regulation of fever generation	8	10	0.002070466	6	10	0.058959971
GO:2001179	regulation of interleukin-10 secretion	7	8	0.002070466	6	10	0.058959971
GO:0045766	positive regulation of angiogenesis	49	137	0.00217924	45	161	0.113312427
GO:0045667	regulation of osteoblast differentiation	38	99	0.00217924	36	121	0.083444409
GO:1903901	negative regulation of viral life cycle	22	48	0.002726302	20	56	0.05634156
GO:0043032	positive regulation of macrophage activation	14	25	0.002825127	11	27	0.09350392
GO:0051783	regulation of nuclear division	39	104	0.003053502	41	140	0.073914267
GO:1901564	organonitrogen compound metabolic process	830	3422	0.003156399	930	4238	0.100061118
GO:0032886	regulation of microtubule-based process	49	139	0.003156399	46	175	0.204057692
GO:0045190	isotype switching	15	28	0.003210709	14	36	0.069433224
GO:0043371	negative regulation of CD4-positive, alpha-beta T cell differentiation	10	15	0.003210709	9	20	0.085863679
GO:1904467	regulation of tumor necrosis factor secretion	15	28	0.003210709	11	29	0.145588868
GO:0002208	somatic diversification of immunoglobulins involved in immune respon	15	28	0.003210709	14	36	0.069433224
GO:0002204	somatic recombination of immunoglobulin genes involved in immune r	15	28	0.003210709	14	36	0.069433224
GO:0048525	negative regulation of viral process	25	58	0.003317912	23	67	0.055905543
GO:0071902	positive regulation of protein serine/threonine kinase activity	63	190	0.003317912	57	229	0.28845782
GO:0007052	mitotic spindle organization	24	55	0.003420805	25	76	0.065669619
GO:0048634	regulation of muscle organ development	41	112	0.003846792	38	137	0.164280913
GO:1901861	regulation of muscle tissue development	41	112	0.003846792	39	137	0.119319955
GO:0045623	negative regulation of T-helper cell differentiation	9	13	0.0042475	8	17	0.090802077
GO:0042462	eye photoreceptor cell development	11	18	0.004557841	9	26	0.280759343
GO:0043271	negative regulation of ion transport	39	106	0.004557841	32	125	0.359202761
GO:0045671	negative regulation of osteoclast differentiation	11	18	0.004557841	10	22	0.061188208
GO:0070997	neuron death	79	253	0.005058511	78	312	0.190429181
GO:0016447	somatic recombination of immunoglobulin gene segments	15	29	0.005058511	14	39	0.125788709
GO:0090594	inflammatory response to wounding	8	11	0.005308247	7	13	0.063747488
GO:0045342	MHC class II biosynthetic process	8	11	0.005308247	6	10	0.058959971
GO:0051712	positive regulation of killing of cells of other organism	8	11	0.005308247	7	13	0.063747488
GO:0034695	response to prostaglandin E	8	11	0.005308247	6	12	0.13327007
GO:0072577	endothelial cell apoptotic process	20	44	0.005387949	14	49	0.386252655
GO:0016444	somatic cell DNA recombination	17	35	0.005387949	16	47	0.13753744
GO:0002562	somatic diversification of immune receptors via germline recombinatio	17	35	0.005387949	16	47	0.13753744
GO:0002486	antigen processing and presentation of endogenous peptide antigen vi	12	21	0.005610405	11	25	0.058959971
GO:0042226	interleukin-6 biosynthetic process	12	21	0.005610405	11	25	0.058959971
GO:0002677	negative regulation of chronic inflammatory response	5	5	0.005610405	3	4	0.158860232
GO:0031536	positive regulation of exit from mitosis	5	5	0.005610405	4	6	0.117133853
GO:0060558	regulation of calcidiol 1-monooxygenase activity	5	5	0.005610405	4	6	0.117133853
GO:1904018	positive regulation of vasculature development	51	150	0.005816079	48	178	0.158652611
GO:0014706	striated muscle tissue development	88	289	0.005915689	87	359	0.253264158
GO:0060840	artery development	28	70	0.006015093	29	90	0.058626594
GO:0043933	protein-containing complex subunit organization	277	1056	0.006114292	313	1350	0.090303371
GO:0061061	muscle structure development	131	458	0.006200474	131	549	0.190429181
GO:0042976	activation of Janus kinase activity	7	9	0.006358221	6	11	0.091745095
GO:0035739	CD4-positive, alpha-beta T cell proliferation	7	9	0.006358221	6	10	0.058959971
GO:0031622	positive regulation of fever generation	7	9	0.006358221	5	9	0.132273429
GO:2000561	regulation of CD4-positive, alpha-beta T cell proliferation	7	9	0.006358221	6	10	0.058959971
GO:0001936	regulation of endothelial cell proliferation	35	94	0.006429583	27	113	0.48833832
GO:0035740	CD8-positive, alpha-beta T cell proliferation	6	7	0.006871207	5	8	0.083146947
GO:0002829	negative regulation of type 2 immune response	6	7	0.006871207	5	10	0.185837225
GO:2000564	regulation of CD8-positive, alpha-beta T cell proliferation	6	7	0.006871207	5	8	0.083146947
GO:0060556	regulation of vitamin D biosynthetic process	6	7	0.006871207	5	8	0.083146947
GO:0046549	retinal cone cell development	6	7	0.006871207	5	8	0.083146947
GO:0018193	peptidyl-amino acid modification	175	637	0.006952778	179	813	0.443727768
GO:0072376	protein activation cascade	29	74	0.006952778	24	75	0.097038184
GO:0045069	regulation of viral genome replication	21	48	0.006952778	19	61	0.185837225
GO:1901222	regulation of NIK/NF-kappaB signaling	25	61	0.007332348	26	85	0.126935407
GO:2000351	regulation of endothelial cell apoptotic process	19	42	0.007520997	13	46	0.434971698
GO:0016445	somatic diversification of immunoglobulins	16	33	0.007592354	15	42	0.113418421
GO:0002244	hematopoietic progenitor cell differentiation	35	95	0.007841538	35	114	0.061148471
GO:0030149	sphingolipid catabolic process	11	19	0.007841538	7	23	0.443727768
GO:0051155	positive regulation of striated muscle cell differentiation	23	55	0.00833598	20	61	0.117133853
GO:0046514	ceramide catabolic process	9	14	0.008470088	5	15	0.443727768
GO:0002544	chronic inflammatory response	9	14	0.008470088	6	14	0.222301272
GO:0046851	negative regulation of bone remodeling	9	14	0.008470088	7	14	0.092179177

GO:0034694	response to prostaglandin	9	14	0.008470088	7	15	0.130095324
GO:0051279	regulation of release of sequestered calcium ion into cytosol	26	65	0.008751922	22	66	0.081539711
GO:0043412	macromolecule modification	493	1983	0.00900635	529	2503	0.593498796
GO:1901214	regulation of neuron death	71	228	0.009374927	70	284	0.253287989
GO:0016202	regulation of striated muscle tissue development	39	110	0.009455304	37	135	0.190429181
GO:0051402	neuron apoptotic process	59	183	0.009544575	57	218	0.1692
GO:1903522	regulation of blood circulation	53	161	0.009919017	48	192	0.332530728
GO:1903523	negative regulation of blood circulation	13	25	0.010066942	10	33	0.403567704
GO:0071624	positive regulation of granulocyte chemotaxis	13	25	0.010066942	11	25	0.058959971
GO:0055025	positive regulation of cardiac muscle tissue development	18	40	0.010602538	15	49	0.268218337
GO:0002200	somatic diversification of immune receptors	18	40	0.010602538	18	51	0.080350934
GO:0098662	inorganic cation transmembrane transport	115	401	0.010822066	115	449	0.058520301
GO:0003151	outflow tract morphogenesis	23	56	0.010822066	24	71	0.058626594
GO:0051341	regulation of oxidoreductase activity	23	56	0.010822066	20	67	0.219446284
GO:0009725	response to hormone	119	417	0.010822066	124	509	0.150143727
GO:0002523	leukocyte migration involved in inflammatory response	10	17	0.011038701	8	18	0.121544217
GO:2001236	regulation of extrinsic apoptotic signaling pathway	37	104	0.011316011	37	123	0.066383212
GO:0051709	regulation of killing of cells of other organism	8	12	0.011337252	7	14	0.092179177
GO:0045064	T-helper 2 cell differentiation	8	12	0.011337252	6	14	0.222301272
GO:0034162	toll-like receptor 9 signaling pathway	8	12	0.011337252	7	13	0.063747488
GO:0042359	vitamin D metabolic process	8	12	0.011337252	7	14	0.092179177
GO:2000181	negative regulation of blood vessel morphogenesis	28	73	0.011791567	24	96	0.443727768
GO:1904019	epithelial cell apoptotic process	30	80	0.012255601	23	89	0.443727768
GO:0032890	regulation of organic acid transport	21	50	0.012339106	19	59	0.150744054
GO:0019538	protein metabolic process	698	2883	0.012517284	768	3597	0.443727768
GO:0016525	negative regulation of angiogenesis	27	70	0.012836889	23	93	0.443727768
GO:0050890	cognition	65	208	0.012919315	64	240	0.10475
GO:0006925	inflammatory cell apoptotic process	11	20	0.012965641	10	22	0.061188208
GO:0046466	membrane lipid catabolic process	11	20	0.012965641	8	26	0.443727768
GO:0043243	positive regulation of protein complex disassembly	11	20	0.012965641	7	22	0.443727768
GO:0006066	alcohol metabolic process	58	182	0.013666887	63	233	0.086510665
GO:1904062	regulation of cation transmembrane transport	73	239	0.013666887	70	263	0.088819418
GO:0043270	positive regulation of ion transport	68	220	0.013828708	67	247	0.069433224
GO:0051092	positive regulation of NF-kappaB transcription factor activity	33	91	0.013828708	34	110	0.060751604
GO:0098660	inorganic ion transmembrane transport	118	416	0.01389667	120	474	0.065191265
GO:0051781	positive regulation of cell division	23	57	0.01389667	17	56	0.246170094
GO:1901224	positive regulation of NIK/NF-kappaB signaling	18	41	0.014284586	16	62	0.443727768
GO:0002468	dendritic cell antigen processing and presentation	7	10	0.015020809	6	11	0.091745095
GO:1900165	negative regulation of interleukin-6 secretion	7	10	0.015020809	5	10	0.185837225
GO:0033004	negative regulation of mast cell activation	7	10	0.015020809	6	10	0.058959971
GO:0001781	neutrophil apoptotic process	7	10	0.015020809	6	11	0.091745095
GO:0045346	regulation of MHC class II biosynthetic process	7	10	0.015020809	5	9	0.132273429
GO:0010543	regulation of platelet activation	13	26	0.015020809	12	28	0.054913265
GO:0051353	positive regulation of oxidoreductase activity	16	35	0.015190341	14	37	0.086510665
GO:0010506	regulation of autophagy	44	131	0.015268143	45	170	0.193772868
GO:0042461	photoreceptor cell development	14	29	0.01537693	10	40	0.582351356
GO:0045944	positive regulation of transcription by RNA polymerase II	184	686	0.01537693	188	871	0.549509931
GO:0070269	pyroptosis	9	15	0.01537693	8	17	0.090802077
GO:0031650	regulation of heat generation	9	15	0.01537693	7	14	0.092179177
GO:0032069	regulation of nuclease activity	9	15	0.01537693	9	20	0.085863679
GO:0046777	protein autophosphorylation	51	157	0.015634415	49	186	0.190429181
GO:0001938	positive regulation of endothelial cell proliferation	27	71	0.015710859	20	82	0.50289955
GO:0035051	cardiocyte differentiation	42	124	0.015759276	38	147	0.305300213
GO:0032088	negative regulation of NF-kappaB transcription factor activity	21	51	0.015759276	19	55	0.084372148
GO:0007160	cell-matrix adhesion	46	139	0.016810212	49	169	0.055548679
GO:0043523	regulation of neuron apoptotic process	53	165	0.016870352	52	196	0.163200221
GO:0030522	intracellular receptor signaling pathway	38	110	0.017034828	35	144	0.443727768
GO:0043406	positive regulation of MAP kinase activity	50	154	0.017273415	46	181	0.292605519
GO:0051302	regulation of cell division	36	103	0.017511166	34	112	0.074549608
GO:0022411	cellular component disassembly	59	188	0.01773257	56	244	0.464462458
GO:0090068	positive regulation of cell cycle process	48	147	0.018308317	51	183	0.08866578
GO:0002374	cytokine secretion involved in immune response	10	18	0.018373934	8	19	0.158860232
GO:0022617	extracellular matrix disassembly	10	18	0.018373934	8	23	0.319390091
GO:1902932	positive regulation of alcohol biosynthetic process	10	18	0.018373934	9	21	0.114007867
GO:0045624	positive regulation of T-helper cell differentiation	10	18	0.018373934	9	21	0.114007867
GO:0034123	positive regulation of toll-like receptor signaling pathway	10	18	0.018373934	9	21	0.114007867
GO:0051023	regulation of immunoglobulin secretion	10	18	0.018373934	8	19	0.158860232
GO:1901989	positive regulation of cell cycle phase transition	22	55	0.018887584	22	72	0.168974564
GO:0003263	cardioblast proliferation	6	8	0.018906821	5	10	0.185837225
GO:0071380	cellular response to prostaglandin E stimulus	6	8	0.018906821	4	9	0.343598605
GO:2001187	positive regulation of CD8-positive, alpha-beta T cell activation	6	8	0.018906821	5	9	0.132273429
GO:0045348	positive regulation of MHC class II biosynthetic process	6	8	0.018906821	4	7	0.185837225
GO:0010881	regulation of cardiac muscle contraction by regulation of the release of	6	8	0.018906821	5	9	0.132273429
GO:0003264	regulation of cardioblast proliferation	6	8	0.018906821	5	10	0.185837225
GO:0002604	regulation of dendritic cell antigen processing and presentation	6	8	0.018906821	5	9	0.132273429
GO:0044057	regulation of system process	111	392	0.018906821	111	465	0.239579319
GO:2000551	regulation of T-helper 2 cell cytokine production	6	8	0.018906821	5	9	0.132273429
GO:0042670	retinal cone cell differentiation	6	8	0.018906821	5	9	0.132273429
GO:0042368	vitamin D biosynthetic process	6	8	0.018906821	5	10	0.185837225
GO:0003007	heart morphogenesis	58	185	0.019260153	63	227	0.057609481
GO:0043405	regulation of MAP kinase activity	63	204	0.019260153	64	239	0.09742473
GO:0007051	spindle organization	31	86	0.019573866	33	117	0.175434971
GO:0045911	positive regulation of DNA recombination	11	21	0.020128831	10	28	0.213566775
GO:0060343	trabecula formation	11	21	0.020128831	10	23	0.080350934
GO:0043569	negative regulation of insulin-like growth factor receptor signaling path	4	4	0.020439098	2	6	0.593498796
GO:0030886	negative regulation of myeloid dendritic cell activation	4	4	0.020439098	3	4	0.158860232
GO:2000566	positive regulation of CD8-positive, alpha-beta T cell proliferation	4	4	0.020439098	3	4	0.158860232
GO:0072513	positive regulation of secondary heart field cardioblast proliferation	4	4	0.020439098	3	4	0.158860232
GO:0002666	positive regulation of T cell tolerance induction	4	4	0.020439098	4	5	0.058959971
GO:0051136	regulation of NK T cell differentiation	4	4	0.020439098	4	5	0.058959971
GO:0055024	regulation of cardiac muscle tissue development	26	69	0.020745088	22	85	0.443727768
GO:0045019	negative regulation of nitric oxide biosynthetic process	8	13	0.021096854	7	16	0.167441784
GO:1904406	negative regulation of nitric oxide metabolic process	8	13	0.021096854	7	16	0.167441784
GO:0031652	positive regulation of heat generation	8	13	0.021096854	6	12	0.13327007
GO:0002830	positive regulation of type 2 immune response	8	13	0.021096854	7	14	0.092179177
GO:0055023	positive regulation of cardiac muscle tissue growth	14	30	0.021230479	12	38	0.301565136
GO:0032768	regulation of monooxygenase activity	14	30	0.021230479	9	31	0.443727768
GO:0031326	regulation of cellular biosynthetic process	460	1864	0.021412438	487	2359	0.768244881
GO:0043615	astrocyte cell migration	5	6	0.021548293	3	8	0.460625
GO:0009120	deoxyribonucleoside metabolic process	5	6	0.021548293	4	6	0.117133853
GO:0002925	positive regulation of humoral immune response mediated by circulati	5	6	0.021548293	4	6	0.117133853

GO:2001181	positive regulation of interleukin-10 secretion	5	6	0.021548293	4	6	0.117133853
GO:0031666	positive regulation of lipopolysaccharide-mediated signaling pathway	5	6	0.021548293	5	8	0.083146947
GO:0044087	regulation of cellular component biogenesis	149	548	0.021548293	155	682	0.358339608
GO:0071640	regulation of macrophage inflammatory protein 1 alpha production	5	6	0.021548293	4	6	0.117133853
GO:0060753	regulation of mast cell chemotaxis	5	6	0.021548293	3	6	0.35647213
GO:0001993	regulation of systemic arterial blood pressure by norepinephrine-epine	5	6	0.021548293	4	5	0.058959971
GO:0051258	protein polymerization	50	156	0.021613599	53	188	0.06627
GO:0060041	retina development in camera-type eye	35	101	0.021844286	33	118	0.186489593
GO:0071356	cellular response to tumor necrosis factor	38	112	0.022570657	40	132	0.050579589
GO:0051054	positive regulation of DNA metabolic process	44	134	0.022634976	44	165	0.190429181
GO:0051495	positive regulation of cytoskeleton organization	41	123	0.022763301	38	158	0.443727768
GO:0002040	sprouting angiogenesis	29	80	0.023037288	27	97	0.247851563
GO:0019079	viral genome replication	22	56	0.023037288	20	71	0.319390091
GO:0140253	cell-cell fusion	18	43	0.024150169	18	54	0.127969844
GO:0000768	syncytium formation by plasma membrane fusion	18	43	0.024150169	18	54	0.127969844
GO:0060976	coronary vasculature development	21	53	0.025177458	20	61	0.117133853
GO:1903426	regulation of reactive oxygen species biosynthetic process	26	70	0.025239164	25	83	0.158652611
GO:1901343	negative regulation of vasculature development	30	84	0.025300771	27	108	0.443727768
GO:0001823	mesonephros development	28	77	0.025423885	27	89	0.127324415
GO:0002726	positive regulation of T cell cytokine production	9	16	0.025423885	8	19	0.158860232
GO:0051043	regulation of membrane protein ectodomain proteolysis	9	16	0.025423885	8	19	0.158860232
GO:0061383	trabecula morphogenesis	16	37	0.027151483	17	48	0.090217969
GO:0031638	zymogen activation	20	50	0.027189411	15	50	0.3028875
GO:0010720	positive regulation of cell development	109	389	0.027470318	115	480	0.211154694
GO:0008630	intrinsic apoptotic signaling pathway in response to DNA damage	25	67	0.027610437	26	87	0.158652611
GO:0003205	cardiac chamber development	42	128	0.027647486	46	160	0.071124508
GO:0009889	regulation of biosynthetic process	469	1910	0.027706355	500	2412	0.74025
GO:0098655	cation transmembrane transport	124	450	0.028227057	125	502	0.09022767
GO:0003156	regulation of animal organ formation	15	34	0.028254236	14	35	0.058959971
GO:2000191	regulation of fatty acid transport	10	19	0.028254236	8	23	0.319390091
GO:0048538	thymus development	15	34	0.028254236	15	42	0.113418421
GO:0071379	cellular response to prostaglandin stimulus	7	11	0.029091556	5	12	0.310898314
GO:0001771	immunological synapse formation	7	11	0.029091556	7	15	0.130095324
GO:0002551	mast cell chemotaxis	7	11	0.029091556	4	11	0.443727768
GO:0034134	toll-like receptor 2 signaling pathway	7	11	0.029091556	7	14	0.092179177
GO:0045840	positive regulation of mitotic nuclear division	14	31	0.029205293	15	44	0.157149289
GO:0060632	regulation of microtubule-based movement	11	22	0.029636493	11	25	0.058959971
GO:0010628	positive regulation of gene expression	279	1097	0.029861212	284	1388	0.801455307
GO:0010831	positive regulation of myotube differentiation	13	28	0.029893964	12	29	0.066830073
GO:0001935	endothelial cell proliferation	35	103	0.029968472	27	124	0.663845856
GO:0002832	negative regulation of response to biotic stimulus	12	25	0.029968472	10	32	0.35647213
GO:0070507	regulation of microtubule cytoskeleton organization	38	114	0.030259753	33	147	0.593498796
GO:0014855	striated muscle cell proliferation	21	54	0.031335948	18	66	0.396889273
GO:0046330	positive regulation of JNK cascade	31	89	0.032282306	30	114	0.340204057
GO:0043433	negative regulation of DNA-binding transcription factor activity	34	100	0.033406278	33	115	0.152061014
GO:0072163	mesonephric epithelium development	27	75	0.033408908	26	86	0.140454509
GO:0072164	mesonephric tubule development	27	75	0.033408908	26	86	0.140454509
GO:0034763	negative regulation of transmembrane transport	27	75	0.033408908	22	92	0.532603077
GO:2001238	positive regulation of extrinsic apoptotic signaling pathway	17	41	0.033408908	17	45	0.053557517
GO:0001657	ureteric bud development	27	75	0.033408908	26	86	0.140454509
GO:0031214	biomineral tissue development	37	111	0.033539038	37	124	0.074679479
GO:0042692	muscle cell differentiation	79	272	0.033824485	75	315	0.35647213
GO:0061138	morphogenesis of a branching epithelium	50	160	0.035791577	53	185	0.053757587
GO:0051149	positive regulation of muscle cell differentiation	24	65	0.036815964	22	71	0.157428985
GO:1904035	regulation of epithelial cell apoptotic process	24	65	0.036815964	18	73	0.500803811
GO:0046328	regulation of JNK cascade	39	119	0.036942623	40	148	0.190429181
GO:1901992	positive regulation of mitotic cell cycle phase transition	19	48	0.037069091	20	62	0.132888874
GO:2000116	regulation of cysteine-type endopeptidase activity	47	149	0.037349705	48	179	0.163327887
GO:0051129	negative regulation of cellular component organization	121	442	0.037861225	130	530	0.117133853
GO:0003197	endocardial cushion development	15	35	0.03790966	13	42	0.3028875
GO:0046165	alcohol biosynthetic process	31	90	0.038314053	30	112	0.292775775
GO:0007254	JNK cascade	41	127	0.039906153	42	156	0.190429181
GO:0061024	membrane organization	108	390	0.039906153	114	460	0.121544217
GO:0001649	osteoblast differentiation	45	142	0.039906153	42	166	0.339737491
GO:0030501	positive regulation of bone mineralization	14	32	0.039906153	13	36	0.139868379
GO:0070542	response to fatty acid	14	32	0.039906153	12	40	0.35647213
GO:0016032	viral process	45	142	0.039906153	49	190	0.236533832
GO:0030048	actin filament-based movement	27	76	0.040077677	24	92	0.406237762
GO:2000317	negative regulation of T-helper 17 type immune response	6	9	0.040077677	5	10	0.185837225
GO:0060340	positive regulation of type I interferon-mediated signaling pathway	6	9	0.040077677	5	10	0.185837225
GO:0007096	regulation of exit from mitosis	6	9	0.040077677	5	10	0.185837225
GO:1904396	regulation of neuromuscular junction development	6	9	0.040077677	5	13	0.358583756
GO:0006949	syncytium formation	18	45	0.040077677	19	56	0.097879052
GO:2001251	negative regulation of chromosome organization	23	62	0.040123705	22	92	0.532603077
GO:0031100	animal organ regeneration	9	17	0.040203811	8	23	0.319390091
GO:0050832	defense response to fungus	9	17	0.040203811	9	20	0.085863679
GO:0002009	morphogenesis of an epithelium	104	374	0.040203811	111	444	0.10699485
GO:0090382	phagosome maturation	9	17	0.040203811	8	18	0.121544217
GO:1904469	positive regulation of tumor necrosis factor secretion	9	17	0.040203811	7	18	0.253233702
GO:0032303	regulation of icosanoid secretion	9	17	0.040203811	8	19	0.158860232
GO:1901739	regulation of myoblast fusion	9	17	0.040203811	8	17	0.090802077
GO:1903514	release of sequestered calcium ion into cytosol by endoplasmic reticulu	9	17	0.040203811	9	20	0.085863679
GO:0046425	regulation of JAK-STAT cascade	35	105	0.040625535	34	109	0.055271148
GO:0010556	regulation of macromolecule biosynthetic process	434	1770	0.041106049	453	2244	0.894185021
GO:0045935	positive regulation of nucleobase-containing compound metabolic pro	253	994	0.041150779	260	1272	0.802647453
GO:0008016	regulation of heart contraction	39	120	0.041944453	34	144	0.469476115
GO:0048844	artery morphogenesis	20	52	0.042053965	21	68	0.168439504
GO:0034109	homotypic cell-cell adhesion	20	52	0.042053965	21	60	0.058959971
GO:0000266	mitochondrial fission	10	20	0.042053965	9	25	0.243466097
GO:0045862	positive regulation of proteolysis	60	200	0.042053965	59	233	0.228934116
GO:0006471	protein ADP-ribosylation	10	20	0.042053965	9	23	0.175521624
GO:0010737	protein kinase A signaling	10	20	0.042053965	7	22	0.443727768
GO:0060038	cardiac muscle cell proliferation	17	42	0.042339768	15	53	0.381365647
GO:0071398	cellular response to fatty acid	11	23	0.042339768	8	28	0.451120707
GO:0006270	DNA replication initiation	11	23	0.042339768	11	25	0.058959971
GO:0042036	negative regulation of cytokine biosynthetic process	11	23	0.042339768	8	25	0.402827677
GO:1901215	negative regulation of neuron death	48	154	0.042339768	44	184	0.443727768
GO:0046530	photoreceptor cell differentiation	17	42	0.042339768	14	54	0.460625
GO:0060045	positive regulation of cardiac muscle cell proliferation	11	23	0.042339768	10	25	0.13121157
GO:2001233	regulation of apoptotic signaling pathway	72	247	0.042339768	77	304	0.163200221
GO:0002026	regulation of the force of heart contraction	11	23	0.042339768	10	27	0.188107404

GO:0060047	heart contraction	45	143	0.044382542	41	173	0.443727768
GO:0043113	receptor clustering	16	39	0.045722549	17	53	0.183678996
GO:0048661	positive regulation of smooth muscle cell proliferation	27	77	0.04712804	29	89	0.050134527
GO:0044070	regulation of anion transport	27	77	0.04712804	28	93	0.127418151
GO:0034766	negative regulation of ion transmembrane transport	21	56	0.047610946	15	73	0.761914807
GO:0044089	positive regulation of cellular component biogenesis	85	300	0.047868747	88	386	0.443727768
GO:0060043	regulation of cardiac muscle cell proliferation	15	36	0.049111909	14	43	0.209901001
GO:0007611	learning or memory	57	190	0.0499506	58	214	0.100137746

RAS_BOS Day 40 GO Terms

GO_Id	goName	RAS_Day40_countDE	RAS_Day40_countAll	RAS_Day40_pv_fdr	BOS_Day40_countDE	BOS_Day40_countAll	BOS_Day40_pv_fdr
GO:0006910	phagocytosis, recognition	23	30	7.77E-09	10	30	0.379469432
GO:0008037	cell recognition	46	92	6.09E-08	37	115	0.082558793
GO:0006958	complement activation, classical pathway	24	43	2.26691E-05	15	45	0.260834303
GO:0045667	regulation of osteoblast differentiation	41	99	0.000130825	39	121	0.071225548
GO:0045669	positive regulation of osteoblast differentiation	25	50	0.000160056	22	60	0.069617467
GO:0006956	complement activation	29	63	0.00024822	23	64	0.074019702
GO:0045063	T-helper 1 cell differentiation	12	17	0.000352953	10	20	0.050989342
GO:0045071	negative regulation of viral genome replication	19	35	0.000395372	16	40	0.071225548
GO:0038061	NIK/NF-kappaB signaling	28	64	0.000909929	30	89	0.076647399
GO:0072376	protein activation cascade	31	74	0.00102621	25	75	0.131777674
GO:0031664	regulation of lipopolysaccharide-mediated signaling pathway	10	14	0.001321653	9	18	0.069617467
GO:0002887	negative regulation of myeloid leukocyte mediated immunity	6	6	0.001358016	5	7	0.057732774
GO:0046639	negative regulation of alpha-beta T cell differentiation	12	19	0.001479744	11	23	0.05225514
GO:0051709	regulation of killing of cells of other organism	9	12	0.001601335	7	14	0.126656794
GO:0072608	interleukin-10 secretion	8	10	0.001848481	7	12	0.057732774
GO:0031620	regulation of fever generation	8	10	0.001848481	6	10	0.074578767
GO:0045625	regulation of T-helper 1 cell differentiation	8	10	0.001848481	7	12	0.057732774
GO:0048304	positive regulation of isotype switching to IgG isotypes	7	8	0.00196752	6	10	0.074578767
GO:2001179	regulation of interleukin-10 secretion	7	8	0.00196752	6	10	0.074578767
GO:1903901	negative regulation of viral life cycle	22	48	0.002203826	20	56	0.111421285
GO:0002446	neutrophil mediated immunity	14	25	0.002450387	11	27	0.14688536
GO:0048525	negative regulation of viral process	25	58	0.002694416	23	67	0.119453663
GO:0044085	cellular component biogenesis	420	1676	0.002877159	529	2130	0.076972966
GO:0043371	negative regulation of CD4-positive, alpha-beta T cell differentiation	10	15	0.002877159	9	20	0.12631564
GO:0045730	respiratory burst	12	20	0.002877159	11	24	0.069617467
GO:0006953	acute-phase response	17	34	0.003096344	13	32	0.10475903
GO:0006996	organelle organization	468	1891	0.003769404	603	2452	0.098290363
GO:0045623	negative regulation of T-helper cell differentiation	9	13	0.003862802	8	17	0.127400608
GO:0045830	positive regulation of isotype switching	9	13	0.003862802	8	17	0.127400608
GO:0045624	positive regulation of T-helper cell differentiation	11	18	0.004079592	9	21	0.165236842
GO:1990774	tumor necrosis factor secretion	15	29	0.004278478	12	31	0.169064523
GO:0061061	muscle structure development	130	458	0.004392102	150	549	0.055727469
GO:0032648	regulation of interferon-beta production	16	32	0.00448948	16	39	0.058519426
GO:0001771	immunological synapse formation	8	11	0.004917449	7	15	0.171956292
GO:0046642	negative regulation of alpha-beta T cell proliferation	8	11	0.004917449	7	12	0.057732774
GO:0043312	neutrophil degranulation	8	11	0.004917449	7	12	0.057732774
GO:0051712	positive regulation of killing of cells of other organism	8	11	0.004917449	7	13	0.086171811
GO:0034695	response to prostaglandin E	8	11	0.004917449	7	12	0.057732774
GO:0002360	T cell lineage commitment	12	21	0.004917449	11	23	0.05225514
GO:0072540	T-helper 17 cell lineage commitment	8	11	0.004917449	7	12	0.057732774
GO:0035745	T-helper 2 cell cytokine production	8	11	0.004917449	7	12	0.057732774
GO:0034104	negative regulation of tissue remodeling	10	16	0.005627815	10	20	0.050989342
GO:0032480	negative regulation of type I interferon production	10	16	0.005627815	10	22	0.091924327
GO:0001780	neutrophil homeostasis	10	16	0.005627815	9	18	0.069617467
GO:0045069	regulation of viral genome replication	21	48	0.005733915	18	61	0.389162842
GO:0031622	positive regulation of fever generation	7	9	0.005925447	5	9	0.165798002
GO:1901222	regulation of NIK/NF-kappaB signaling	25	61	0.005925447	27	85	0.176720376
GO:0001649	osteoblast differentiation	48	142	0.006323141	50	166	0.10475903
GO:0071608	macrophage inflammatory protein-1 alpha production	6	7	0.006451915	5	7	0.057732774
GO:0002829	negative regulation of type 2 immune response	6	7	0.006451915	5	10	0.209213829
GO:0043313	regulation of neutrophil degranulation	6	7	0.006451915	5	8	0.103689861
GO:0033045	regulation of sister chromatid segregation	17	36	0.006451915	21	55	0.053472153
GO:0051770	positive regulation of nitric-oxide synthase biosynthetic process	11	19	0.007023514	9	18	0.069617467
GO:0045191	regulation of isotype switching	11	19	0.007023514	11	25	0.089689355
GO:2000316	regulation of T-helper 17 type immune response	11	19	0.007023514	10	20	0.050989342
GO:0034694	response to prostaglandin	9	14	0.007696684	8	15	0.066065448
GO:0010557	positive regulation of macromolecule biosynthetic process	256	993	0.008558087	320	1265	0.112125
GO:0002832	negative regulation of response to biotic stimulus	13	25	0.008837684	10	32	0.443627816
GO:0045190	isotype switching	14	28	0.009190805	14	36	0.122045563
GO:1901214	regulation of neuron death	70	228	0.009190805	83	284	0.05326456
GO:1904467	regulation of tumor necrosis factor secretion	14	28	0.009190805	11	29	0.202940443
GO:0002208	somatic diversification of immunoglobulins involved in immune respor	14	28	0.009190805	14	36	0.122045563
GO:0002204	somatic recombination of immunoglobulin genes involved in immune r	14	28	0.009190805	14	36	0.122045563
GO:0050832	defense response to fungus	10	17	0.010026008	10	20	0.050989342
GO:0045064	T-helper 2 cell differentiation	8	12	0.010558821	6	14	0.27660124
GO:0043243	positive regulation of protein complex disassembly	11	20	0.011842901	8	22	0.344797724
GO:1901224	positive regulation of NIK/NF-kappaB signaling	18	41	0.0122906	16	62	0.585393023
GO:0007015	actin filament organization	77	258	0.012582538	87	300	0.055693638
GO:0150077	regulation of neuroinflammatory response	12	23	0.012870825	11	24	0.069617467
GO:0010638	positive regulation of organelle organization	88	302	0.01321984	102	409	0.443627816
GO:0016444	somatic cell DNA recombination	16	35	0.01321984	16	47	0.20890641
GO:0002562	somatic diversification of immune receptors via germline recombinatic	16	35	0.01321984	16	47	0.20890641
GO:0010543	regulation of platelet activation	13	26	0.013452891	12	28	0.086171811
GO:0016447	somatic recombination of immunoglobulin gene segments	14	29	0.013611481	14	39	0.198469201
GO:0010632	regulation of epithelial cell migration	49	151	0.013697219	56	183	0.064780379
GO:0061548	ganglion development	7	10	0.014209056	7	13	0.086171811
GO:1900165	negative regulation of interleukin-6 secretion	7	10	0.014209056	5	10	0.209213829
GO:0033004	negative regulation of mast cell activation	7	10	0.014209056	6	10	0.074578767
GO:0001781	neutrophil apoptotic process	7	10	0.014209056	6	11	0.120791407
GO:0043584	nose development	7	10	0.014209056	6	12	0.171956292
GO:0070269	pyroptosis	9	15	0.014209056	8	17	0.127400608
GO:1900225	regulation of NLRP3 inflammasome complex assembly	7	10	0.014209056	7	12	0.057732774
GO:0032069	regulation of nuclease activity	9	15	0.014209056	9	20	0.12631564
GO:0043433	negative regulation of DNA-binding transcription factor activity	35	100	0.014778599	36	115	0.125594479
GO:0043534	blood vessel endothelial cell migration	27	72	0.015705044	27	94	0.356274802
GO:0048738	cardiac muscle tissue development	55	175	0.016174079	64	219	0.098575139
GO:0022617	extracellular matrix disassembly	10	18	0.017027093	10	23	0.122045563
GO:0042462	eye photoreceptor cell development	10	18	0.017027093	9	26	0.371895193
GO:0045671	negative regulation of osteoclast differentiation	10	18	0.017027093	10	22	0.091924327
GO:0051023	regulation of immunoglobulin secretion	10	18	0.017027093	8	19	0.202940443
GO:0048844	artery morphogenesis	21	52	0.017202933	24	68	0.080321671
GO:0043113	receptor clustering	17	39	0.017282421	16	53	0.380247574
GO:0060041	retina development in camera-type eye	35	101	0.017728428	39	118	0.05225514
GO:0010881	regulation of cardiac muscle contraction by regulation of the release o	6	8	0.018431844	5	9	0.165798002
GO:2000551	regulation of T-helper 2 cell cytokine production	6	8	0.018431844	5	9	0.165798002
GO:0051124	synaptic growth at neuromuscular junction	6	8	0.018431844	4	9	0.379469432
GO:0016032	viral process	46	142	0.018610728	52	190	0.286401306
GO:0016445	somatic diversification of immunoglobulins	15	33	0.018822976	15	42	0.182454509
GO:0010506	regulation of autophagy	43	131	0.019257284	46	170	0.366058394

GO:0035909	aorta morphogenesis	12	24	0.019333052	14	32	0.050640013
GO:0044364	disruption of cells of other organism	13	27	0.019577835	12	31	0.169064523
GO:0031640	killing of cells of other organism	13	27	0.019577835	12	31	0.169064523
GO:0019079	viral genome replication	22	56	0.0200087	20	71	0.443627816
GO:0030540	female genitalia development	8	13	0.020287803	8	16	0.091576453
GO:0050711	negative regulation of interleukin-1 secretion	8	13	0.020287803	7	13	0.086171811
GO:0002830	positive regulation of type 2 immune response	8	13	0.020287803	7	14	0.126656794
GO:0043569	negative regulation of insulin-like growth factor receptor signaling patl	4	4	0.020375482	2	6	0.60591003
GO:0043305	negative regulation of mast cell degranulation	4	4	0.020375482	3	4	0.176720376
GO:1902565	positive regulation of neutrophil activation	4	4	0.020375482	3	4	0.176720376
GO:0043315	positive regulation of neutrophil degranulation	4	4	0.020375482	3	4	0.176720376
GO:0002666	positive regulation of T cell tolerance induction	4	4	0.020375482	4	5	0.069617467
GO:0051136	regulation of NK T cell differentiation	4	4	0.020375482	4	5	0.069617467
GO:0009120	deoxyribonucleoside metabolic process	5	6	0.021327486	4	6	0.139103542
GO:0018193	peptidyl-amino acid modification	168	637	0.021327486	214	813	0.069617467
GO:0002925	positive regulation of humoral immune response mediated by circulati	5	6	0.021327486	4	6	0.139103542
GO:2001181	positive regulation of interleukin-10 secretion	5	6	0.021327486	4	6	0.139103542
GO:0031666	positive regulation of lipopolysaccharide-mediated signaling pathway	5	6	0.021327486	5	8	0.103689861
GO:0071640	regulation of macrophage inflammatory protein 1 alpha production	5	6	0.021327486	4	6	0.139103542
GO:0060753	regulation of mast cell chemotaxis	5	6	0.021327486	3	6	0.380247574
GO:0001993	regulation of systemic arterial blood pressure by norepinephrine-epine	5	6	0.021327486	4	5	0.069617467
GO:0002291	T cell activation via T cell receptor contact with antigen bound to MHC	5	6	0.021327486	5	7	0.057732774
GO:0042552	myelination	36	106	0.021469404	40	122	0.054635352
GO:0043271	negative regulation of ion transport	36	106	0.021469404	38	125	0.159731214
GO:0010720	positive regulation of cell development	108	389	0.022241126	131	480	0.076972966
GO:0002200	somatic diversification of immune receptors	17	40	0.022560656	18	51	0.151473578
GO:0002726	positive regulation of T cell cytokine production	9	16	0.024091891	8	19	0.202940443
GO:1900424	regulation of defense response to bacterium	9	16	0.024091891	6	18	0.443627816
GO:0014015	positive regulation of gliogenesis	22	57	0.024831189	20	70	0.433062109
GO:0045687	positive regulation of glial cell differentiation	15	34	0.025543767	10	42	0.701862398
GO:0019538	protein metabolic process	680	2883	0.025698888	868	3597	0.133863151
GO:0044087	regulation of cellular component biogenesis	146	548	0.025942578	182	682	0.067930328
GO:0030149	sphingolipid catabolic process	10	19	0.026647431	8	23	0.380247574
GO:0031032	actomyosin structure organization	46	145	0.027611959	45	168	0.380247574
GO:0043302	positive regulation of leukocyte degranulation	11	22	0.027802993	10	24	0.155890076
GO:0051985	negative regulation of chromosome segregation	12	25	0.027919483	14	33	0.065253846
GO:0043304	regulation of mast cell degranulation	12	25	0.027919483	11	26	0.117698347
GO:0032495	response to muramyl dipeptide	7	11	0.028024175	7	14	0.126656794
GO:0034134	toll-like receptor 2 signaling pathway	7	11	0.028024175	7	14	0.126656794
GO:0007051	spindle organization	30	86	0.029188172	38	117	0.069617467
GO:0008366	axon ensheathment	36	108	0.029477945	40	124	0.069320678
GO:0022411	cellular component disassembly	57	188	0.029477945	69	244	0.145917948
GO:0007272	ensheathment of neurons	36	108	0.029477945	40	124	0.069320678
GO:0032637	interleukin-8 production	20	51	0.029477945	20	57	0.128942424
GO:0051495	positive regulation of cytoskeleton organization	40	123	0.029710223	43	158	0.366203761
GO:1901215	negative regulation of neuron death	48	154	0.032464578	55	184	0.095017704
GO:0065003	protein-containing complex assembly	233	921	0.032522061	299	1197	0.190605959
GO:0061024	membrane organization	107	390	0.032863764	120	460	0.217823864
GO:0003197	endocardial cushion development	15	35	0.033878499	17	42	0.056442936
GO:0060761	negative regulation of response to cytokine stimulus	15	35	0.033878499	17	43	0.069617467
GO:0034394	protein localization to cell surface	15	35	0.033878499	14	43	0.311194858
GO:0031641	regulation of myelination	15	35	0.033878499	14	41	0.247085246
GO:0035051	cardiocyte differentiation	40	124	0.034186773	47	147	0.050817435
GO:0046514	ceramide catabolic process	8	14	0.034575483	6	15	0.338285366
GO:0060841	venous blood vessel development	8	14	0.034575483	4	16	0.701862398
GO:0006949	syncytium formation	18	45	0.035133431	19	56	0.17695022
GO:0002042	cell migration involved in sprouting angiogenesis	14	32	0.03598868	12	45	0.571496537
GO:1903727	positive regulation of phospholipid metabolic process	14	32	0.03598868	14	38	0.172824979
GO:0070542	response to fatty acid	14	32	0.03598868	14	40	0.209556153
GO:2000181	negative regulation of blood vessel morphogenesis	26	73	0.037064792	30	96	0.174632786
GO:0044057	regulation of system process	107	392	0.038103862	124	465	0.153365101
GO:0002523	leukocyte migration involved in inflammatory response	9	17	0.038150742	9	18	0.069617467
GO:0002313	mature B cell differentiation involved in immune response	9	17	0.038150742	9	18	0.069617467
GO:0032303	regulation of icosanoid secretion	9	17	0.038150742	8	19	0.202940443
GO:0033688	regulation of osteoblast proliferation	9	17	0.038150742	9	23	0.222175141
GO:1903514	release of sequestered calcium ion into cytosol by endoplasmic reticulu	9	17	0.038150742	8	20	0.240873318
GO:0042976	activation of Janus kinase activity	6	9	0.038737776	6	11	0.120791407
GO:0035739	CD4-positive, alpha-beta T cell proliferation	6	9	0.038737776	6	10	0.074578767
GO:0072610	interleukin-12 secretion	6	9	0.038737776	5	9	0.165798002
GO:0002315	marginal zone B cell differentiation	6	9	0.038737776	6	10	0.074578767
GO:0050713	negative regulation of interleukin-1 beta secretion	6	9	0.038737776	5	9	0.165798002
GO:2000317	negative regulation of T-helper 17 type immune response	6	9	0.038737776	5	10	0.209213829
GO:0060340	positive regulation of type I interferon-mediated signaling pathway	6	9	0.038737776	5	10	0.209213829
GO:2000561	regulation of CD4-positive, alpha-beta T cell proliferation	6	9	0.038737776	6	10	0.074578767
GO:1904396	regulation of neuromuscular junction development	6	9	0.038737776	4	13	0.587104081
GO:0033006	regulation of mast cell activation involved in immune response	12	26	0.038774409	11	27	0.14688536
GO:0048009	insulin-like growth factor receptor signaling pathway	10	20	0.039364425	11	29	0.202940443
GO:0046466	membrane lipid catabolic process	10	20	0.039364425	9	26	0.371895193
GO:0033687	osteoblast proliferation	10	20	0.039364425	10	26	0.20890641
GO:0006471	protein ADP-ribosylation	10	20	0.039364425	10	23	0.122045563
GO:0061298	retina vasculature development in camera-type eye	10	20	0.039364425	11	23	0.05225514
GO:0042036	negative regulation of cytokine biosynthetic process	11	23	0.03946246	11	25	0.089689355
GO:0043552	positive regulation of phosphatidylinositol 3-kinase activity	11	23	0.03946246	11	26	0.117698347
GO:0032677	regulation of interleukin-8 production	19	49	0.040044588	19	53	0.120791407
GO:0016525	negative regulation of angiogenesis	25	70	0.040415617	29	93	0.182144789
GO:0051258	protein polymerization	48	156	0.040544234	57	188	0.070133296
GO:0048010	vascular endothelial growth factor receptor signaling pathway	15	36	0.044324258	12	41	0.443627816
GO:0051962	positive regulation of nervous system development	104	382	0.044733427	128	468	0.076990556
GO:0008630	intrinsic apoptotic signaling pathway in response to DNA damage	24	67	0.0451774	28	87	0.149801034
GO:0042692	muscle cell differentiation	77	272	0.045664058	82	315	0.338285366
GO:0046330	positive regulation of JNK cascade	30	89	0.047156712	33	114	0.281044772
GO:0007163	establishment or maintenance of cell polarity	43	138	0.047436587	50	177	0.211986405
GO:0046328	regulation of JNK cascade	38	119	0.047798733	43	148	0.202940443
GO:0019058	viral life cycle	34	104	0.048197233	38	130	0.210191429
GO:0140253	cell-cell fusion	17	43	0.048240852	18	54	0.202940443
GO:0000768	syncytium formation by plasma membrane fusion	17	43	0.048240852	18	54	0.202940443
GO:0048143	astrocyte activation	7	12	0.049157015	7	16	0.202940443
GO:0043374	CD8-positive, alpha-beta T cell differentiation	7	12	0.049157015	8	15	0.066065448
GO:0045779	negative regulation of bone resorption	7	12	0.049157015	6	12	0.171956292
GO:0048711	positive regulation of astrocyte differentiation	7	12	0.049157015	5	15	0.46859344
GO:0030949	positive regulation of vascular endothelial growth factor receptor signa	7	12	0.049157015	6	13	0.20890641

GO:1903909	regulation of receptor clustering	7	12	0.049157015	5	17	0.587104081
GO:0034162	toll-like receptor 9 signaling pathway	7	12	0.049157015	7	13	0.086171811
GO:0043506	regulation of JUN kinase activity	23	64	0.04950867	24	75	0.196304595
GO:0051057	positive regulation of small GTPase mediated signal transduction	19	50	0.049587597	21	55	0.053472153
GO:0032890	regulation of organic acid transport	19	50	0.049587597	20	59	0.171277372
GO:0007254	JNK cascade	40	127	0.049626607	44	156	0.259572452
GO:0034754	cellular hormone metabolic process	21	57	0.049859406	21	74	0.434803292
GO:0050918	positive chemotaxis	13	30	0.049859406	13	38	0.271016914
GO:0043551	regulation of phosphatidylinositol 3-kinase activity	13	30	0.049859406	12	35	0.284850394
